# Supplementary material for: Targeted, genome-scale overexpression in Proteobacteria
Source: mBio. 2026 Sep 21;17(10):e02340-25. doi: 10.1128/mbio.02340-25 (PMC13712794; doi:10.1128/mbio.02340-25)

Figure S1

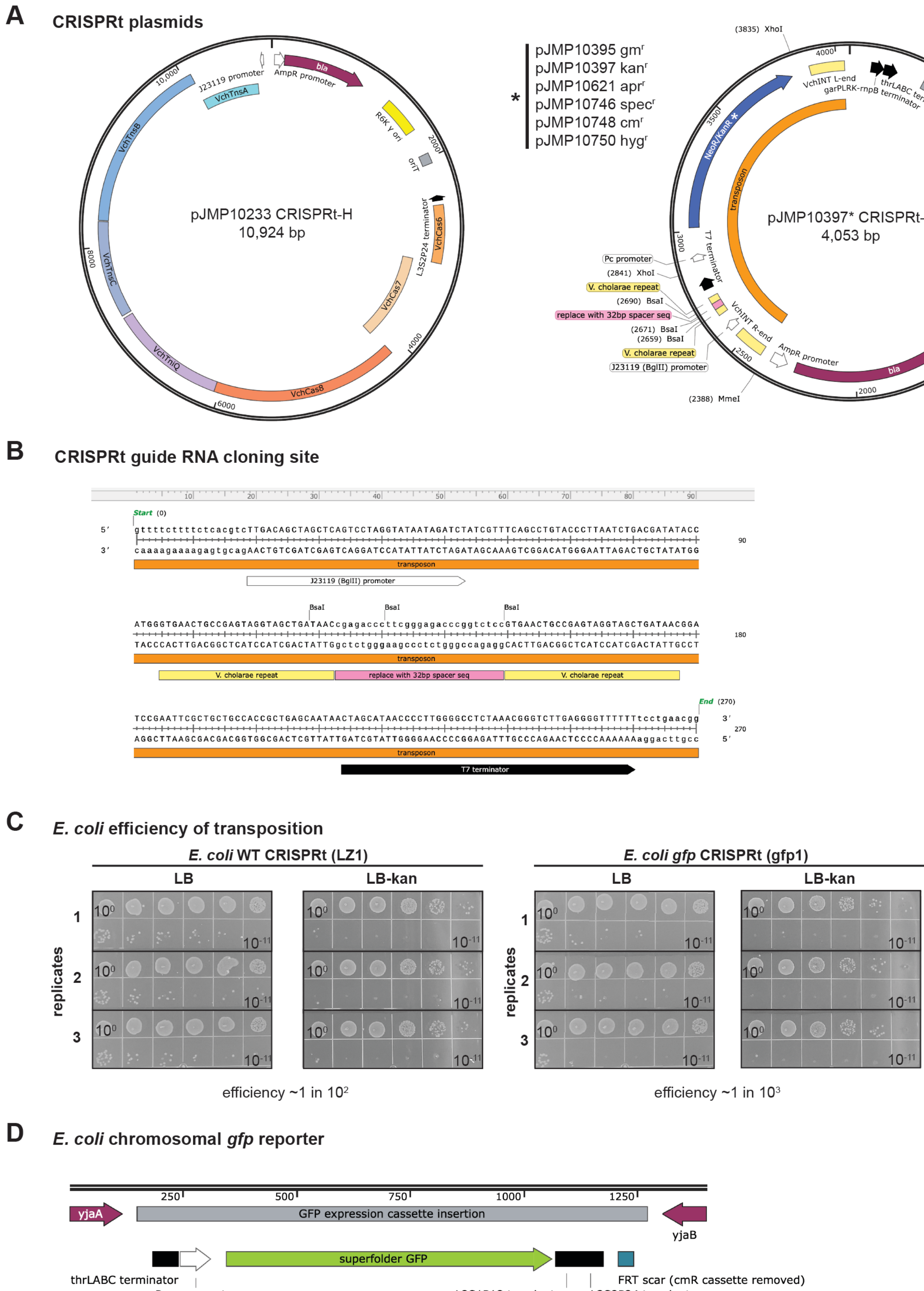

A
CRISPRt plasmids
pJMP10233 CRISPRt-H
10,924 bp
J23119 promoter
VchTnsA
AmpR promoter
bla
R6K γ ori
oriT
L3S2P24 terminator
VchCas6
VchCas7
VchCas8
VchTniQ
VchTnsC
VchTnsB
*
pJMP10395 gm^r
pJMP10397 kan^r
pJMP10621 apr^r
pJMP10746 spec^r
pJMP10748 cm^r
pJMP10750 hyg^r
pJMP10397* CRISPRt-T
4,053 bp
(3835) XhoI
MmeI (429)
VchINT L-end
garPLRK-rnpB terminator
thrLABC terminator
oriT
R6K γ ori
NeoR/KanR *
transposon
T7 terminator
Pc promoter
(2841) XhoI
V. cholarae repeat
(2690) BsaI
replace with 32bp spacer seq
(2671) BsaI
(2659) BsaI
V. cholarae repeat
J23119 (BglII) promoter
VchINT R-end
AmpR promoter
bla
(2388) MmeI
B
CRISPRt guide RNA cloning site
Start (0)
5′ gttttcttttctcacgtcTTGACAGCTAGCTCAGTCCTAGGTATAATAGATCTATCGTTTCAGCCTGTACCCTTAATCTGACGATATACC
3′ caaaagaaaagagtgcagAACTGTCGATCGAGTCAGGATCCATATTATCTAGATAGCAAAGTCGGACATGGGAATTAGACTGCTATATGG
90
transposon
J23119 (BglII) promoter
BsaI
BsaI
BsaI
ATGGGTGAACTGCCGAGTAGGTAGCTGATAACcgagacccttcgggagacccggtctccGTGAACTGCCGAGTAGGTAGCTGATAACGGA
TACCCACTTGACGGCTCATCCATCGACTATTGgctctgggaagccctctgggccagaggCACTTGACGGCTCATCCATCGACTATTGCCT
180
transposon
V. cholarae repeat
replace with 32bp spacer seq
V. cholarae repeat
End (270)
TCCGAATTCGCTGCTGCCACCGCTGAGCAATAACTAGCATAACCCCTTGGGGCCTCTAAACGGGTCTTGAGGGGTTTTTTtcctgaacgg 3′
AGGCTTAAGCGACGACGGTGGCGACTCGTTATTGATCGTATTGGGGAACCCCGGAGATTTGCCCAGAACTCCCCAAAAAAaggacttgcc 5′
270
transposon
T7 terminator
C
E. coli efficiency of transposition
E. coli WT CRISPRt (LZ1)
LB
LB-kan
E. coli gfp CRISPRt (gfp1)
LB
LB-kan
replicates
1
2
3
10^0
10^-11
efficiency ~1 in 10^2
efficiency ~1 in 10^3
D
E. coli chromosomal gfp reporter
250
500
750
1000
1250
yjaA
GFP expression cassette insertion
yjaB
superfolder GFP
thrLABC terminator
Pveg promoter
L3S1P13 terminator
L3S2P24 terminator
FRT scar (cmR cassette removed)

Figure S2

A **CRISPRt insertions into *lacZ* (*LZ1* spacer)**

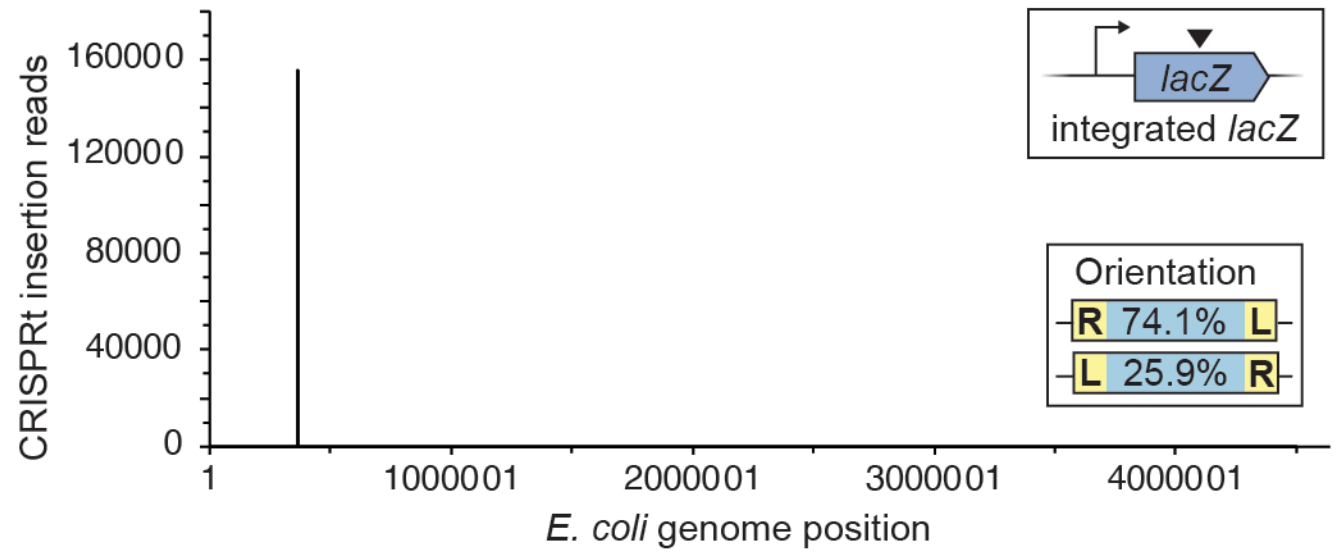

B ***gfp1* insertion position (on target, RL orientation)**

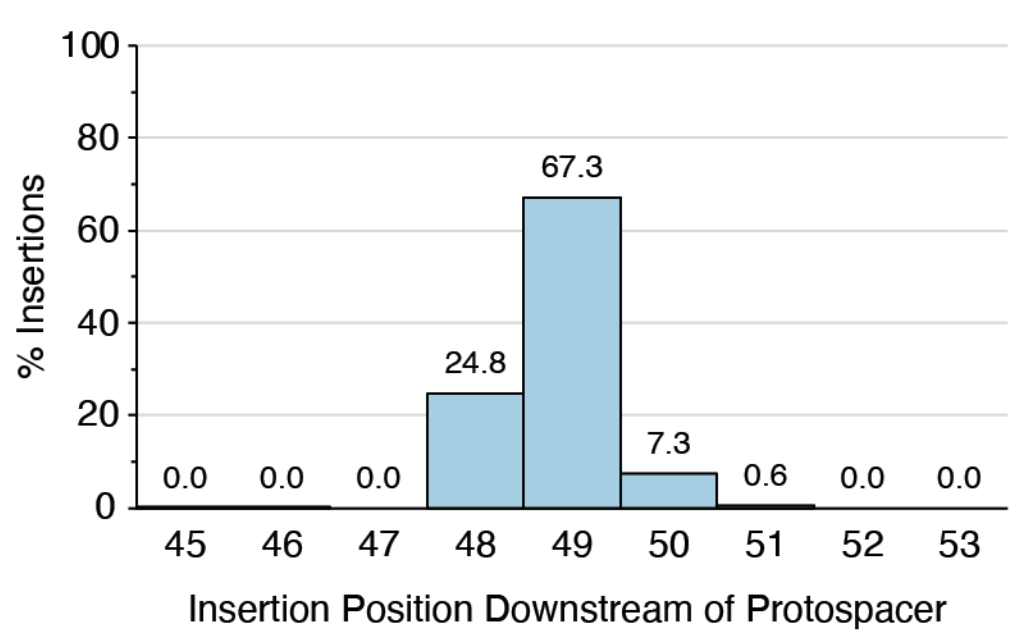

C ***LZ1* insertion position (on target, RL orientation)**

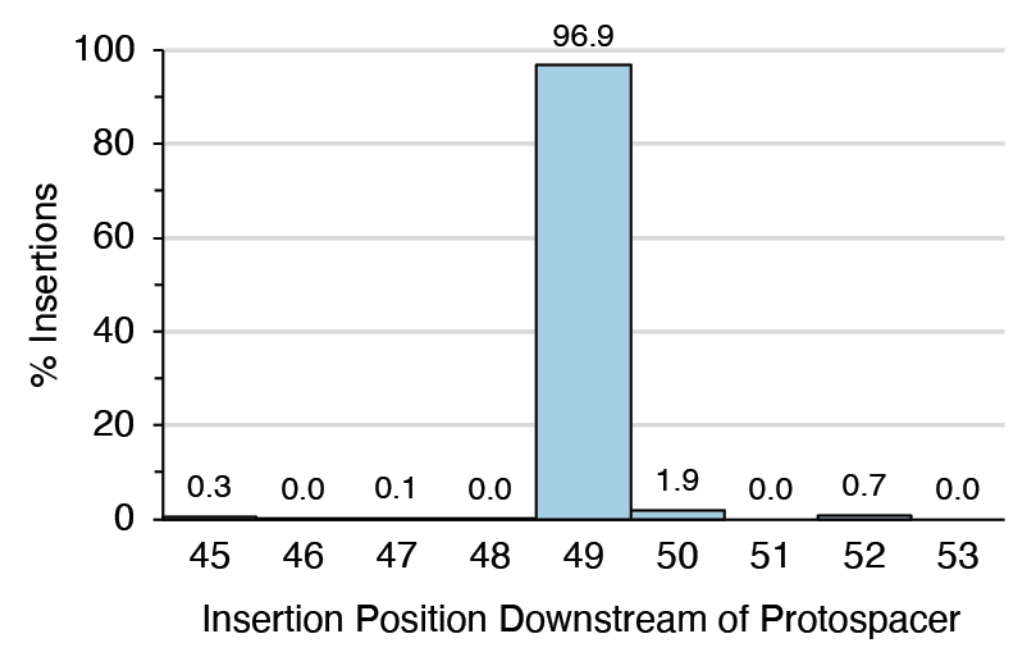

D ***gfp* insertion phenotype**

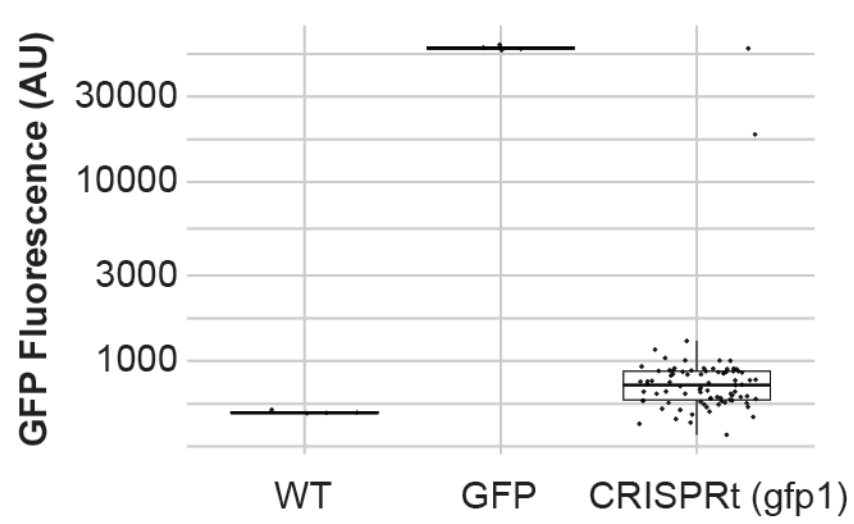

Strain	Parent Strain genotype	CRISPRt guide	Fluorescence phenotype
sJMP163	*E. coli* MG1655 WT	na	white
sJMP2688	*E. coli* MG1655 *gfp*	na	green
isolates	*E. coli* MG1655 *gfp*	gfp1	82/84 white (97.6%)

E ***lacZ* insertion phenotype**

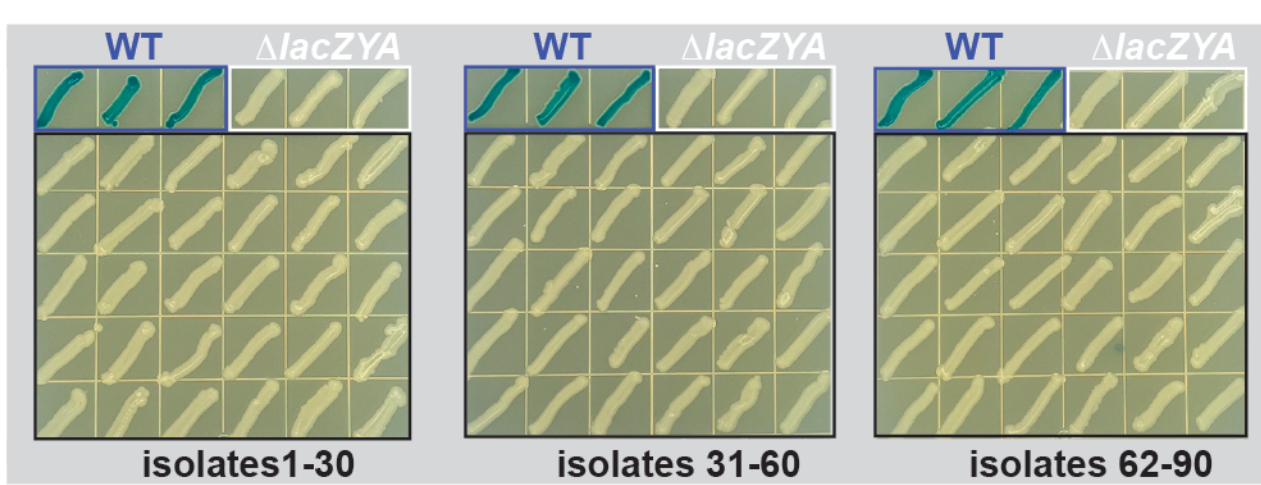

Strain	Parent Strain genotype	CRISPRt guide	X-gal phenotype
sJMP163	*E. coli* MG1655 WT	na	blue
sJMP3272	*E. coli* MG1655 DlacZYA	na	white
isolates	*E. coli* MG1655 WT	LZ1	90/90 white (100%)

F **Frequency of insertion of entire transposon vs transposition only**

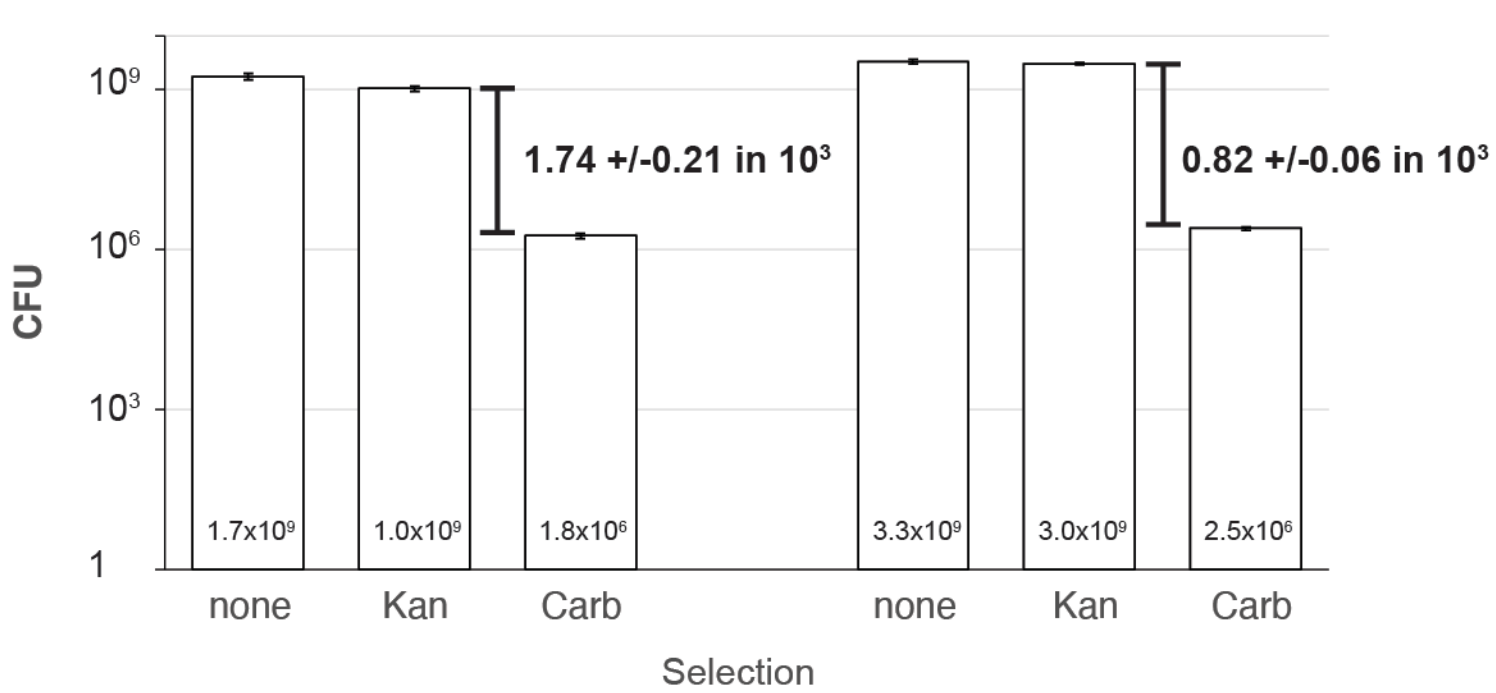

Figure S3

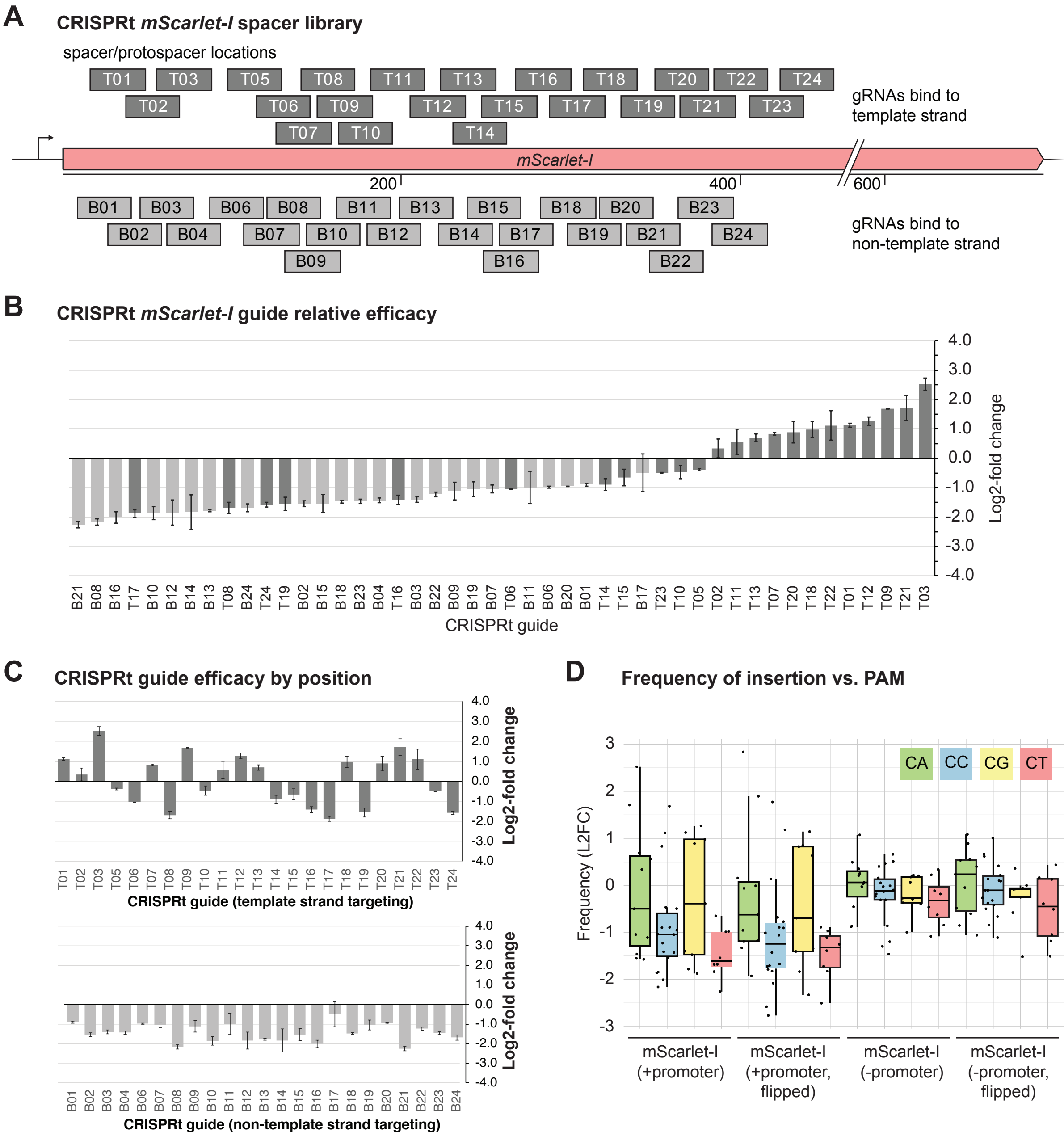

A
CRISPRt mScarlet-I spacer library
spacer/protospacer locations
T01 T03 T05 T08 T11 T13 T16 T18 T20 T22 T24
T02 T06 T09 T12 T15 T17 T19 T21 T23
T07 T10 T14
gRNAs bind to template strand
mScarlet-I
200
400
600
B01 B03 B06 B08 B11 B13 B15 B18 B20 B23
B02 B04 B07 B10 B12 B14 B17 B19 B21 B24
B09 B16 B22
gRNAs bind to non-template strand
B
CRISPRt mScarlet-I guide relative efficacy
Log2-fold change
CRISPRt guide
C
CRISPRt guide efficacy by position
Log2-fold change
CRISPRt guide (template strand targeting)
CRISPRt guide (non-template strand targeting)
D
Frequency of insertion vs. PAM
Frequency (L2FC)
CA
CC
CG
CT
mScarlet-I (+promoter)
mScarlet-I (+promoter, flipped)
mScarlet-I (-promoter)
mScarlet-I (-promoter, flipped)

Figure S4

A

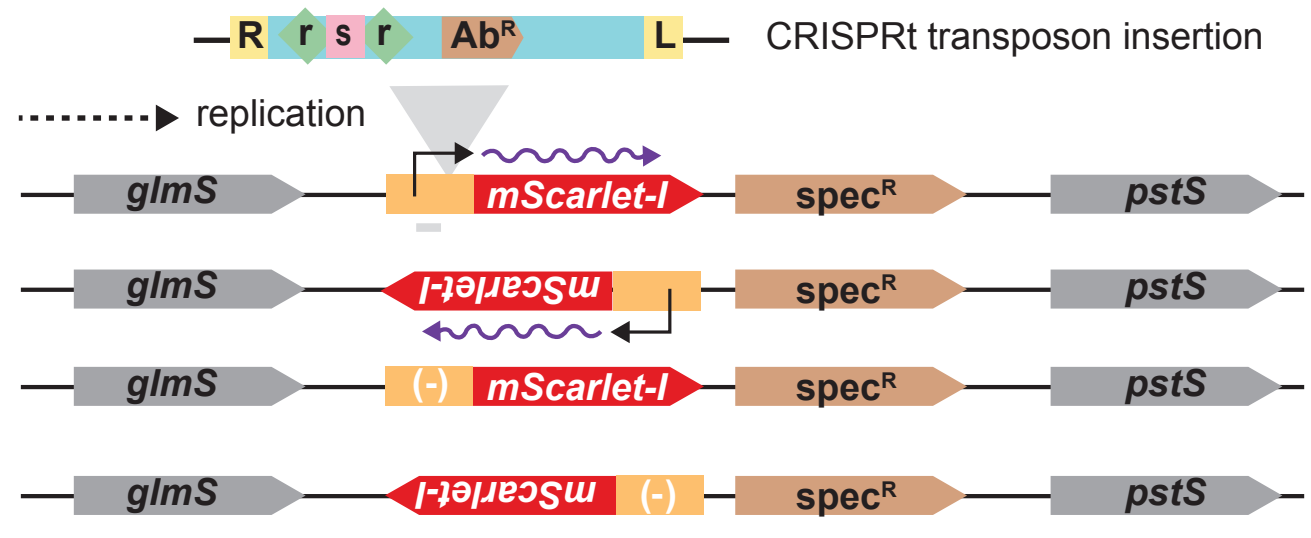

DNA strand	Promoter
leading	+
lagging	+
leading	-
lagging	-

B

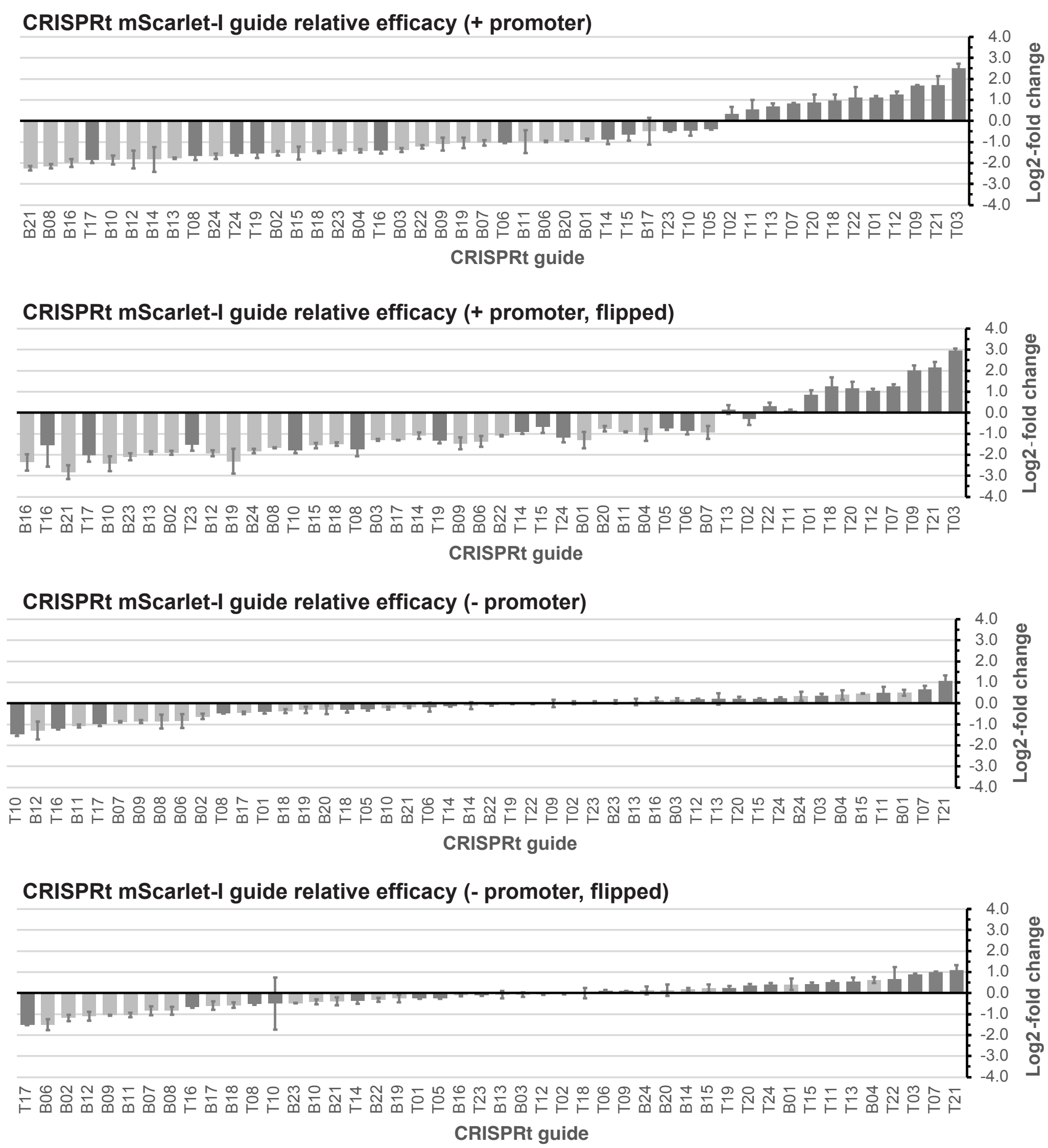

Figure S5

A

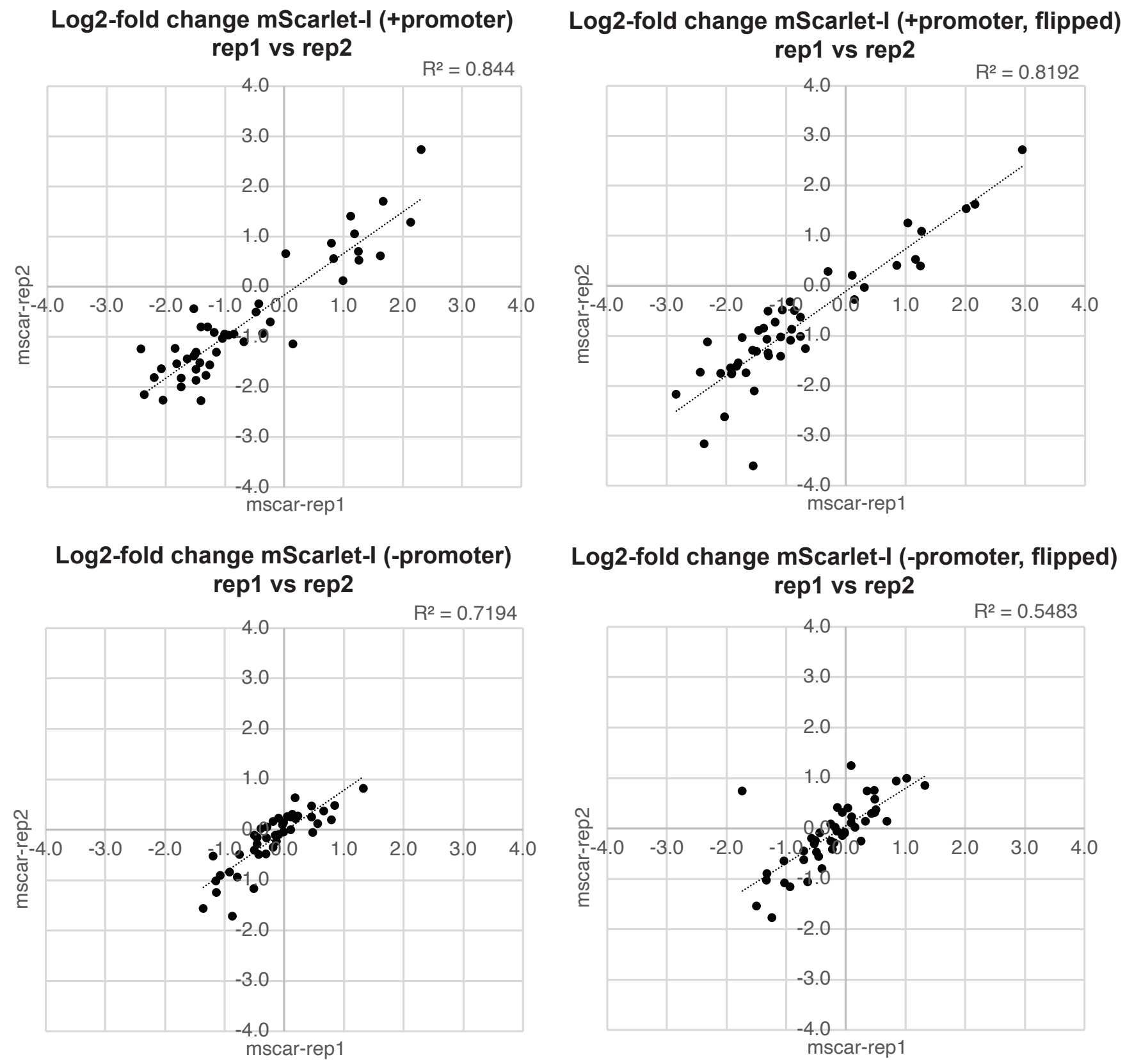
Log2-fold change mScarlet-I (+promoter)
rep1 vs rep2
R² = 0.844
Log2-fold change mScarlet-I (+promoter, flipped)
rep1 vs rep2
R² = 0.8192
Log2-fold change mScarlet-I (-promoter)
rep1 vs rep2
R² = 0.7194
Log2-fold change mScarlet-I (-promoter, flipped)
rep1 vs rep2
R² = 0.5483
mscar-rep1
mscar-rep2
4.0
3.0
2.0
1.0
0.0
-1.0
-2.0
-3.0
-4.0

B

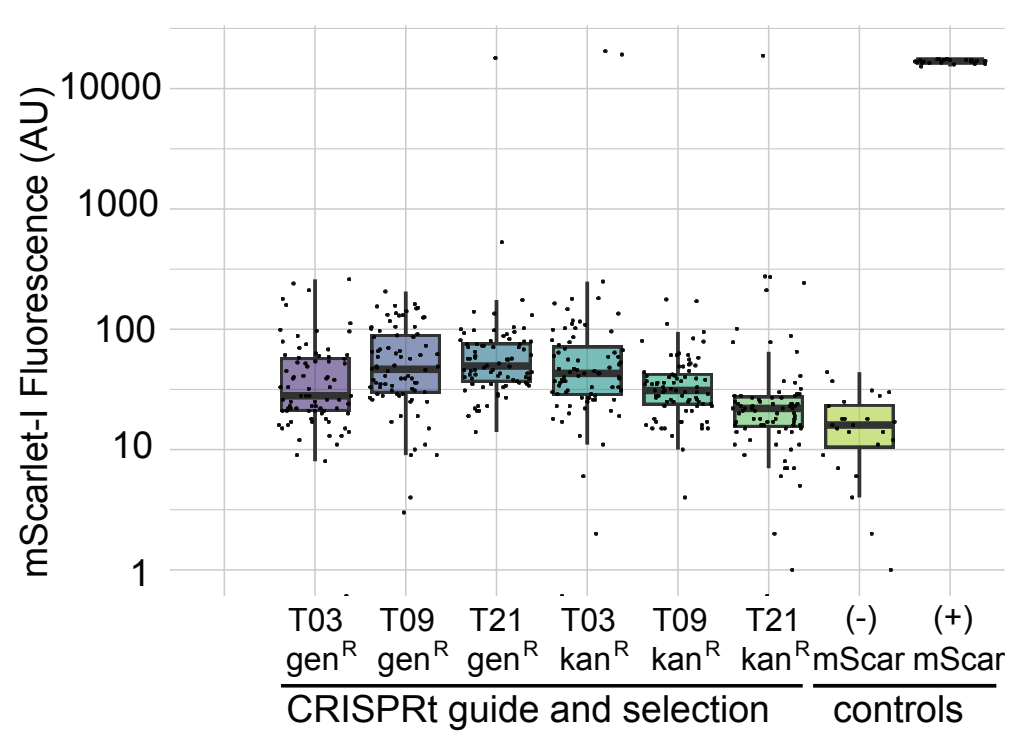
mScarlet-I Fluorescence (AU)
10000
1000
100
10
1
T03 genR
T09 genR
T21 genR
T03 kanR
T09 kanR
T21 kanR
(-) mScar
(+) mScar
CRISPRt guide and selection
controls

Figure S6

A CRISPRtOE Promoter Sequences

A	CTCACTCATTAGGCACCCCAGGC**TTTACA**CTTTATGCTTCCGGCTCG**TATAAT**GTCTAG
H	GGAAAATTTTTTTTCAAAAGTAC**TTGAAA**ATTAATCA•TCCGGCTCG**TATAAT**TCTAGT
V	CACCAAGCCAGTTACCTCGGTTC**TTTAAT**CTTTATGC•TCCGGCTCG**TATAGT**GCTAGC
	nnnnnnnnnnnnnnnnnnnnnnn**TTGACA**nnnnnnnn•nnnnnnnnn**TATAAT**nnnnnn
	-35 spacer -10

B Transposons with outward facing promoters (or no promoter control)

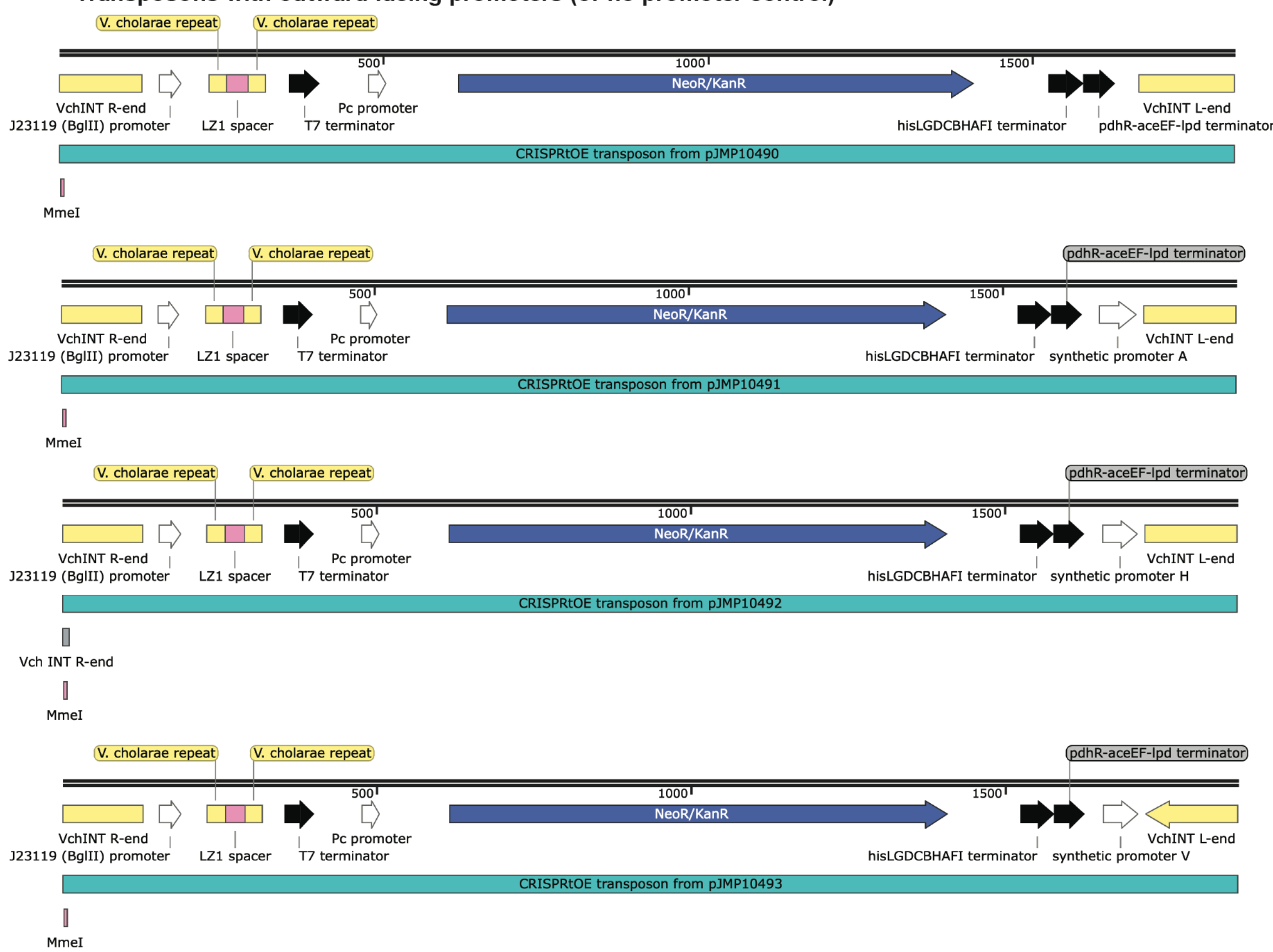

C Chromosomal promoterless mScarlet-I reporter

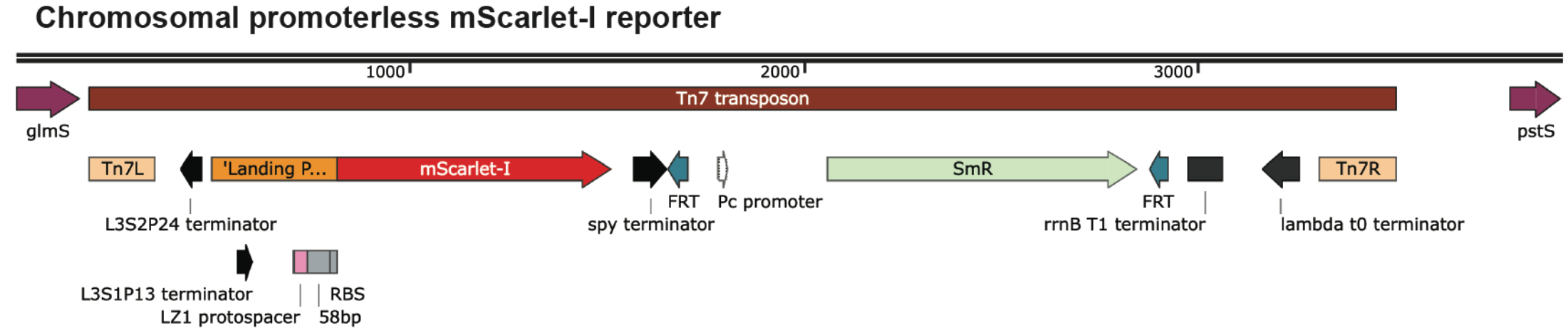

Figure S7

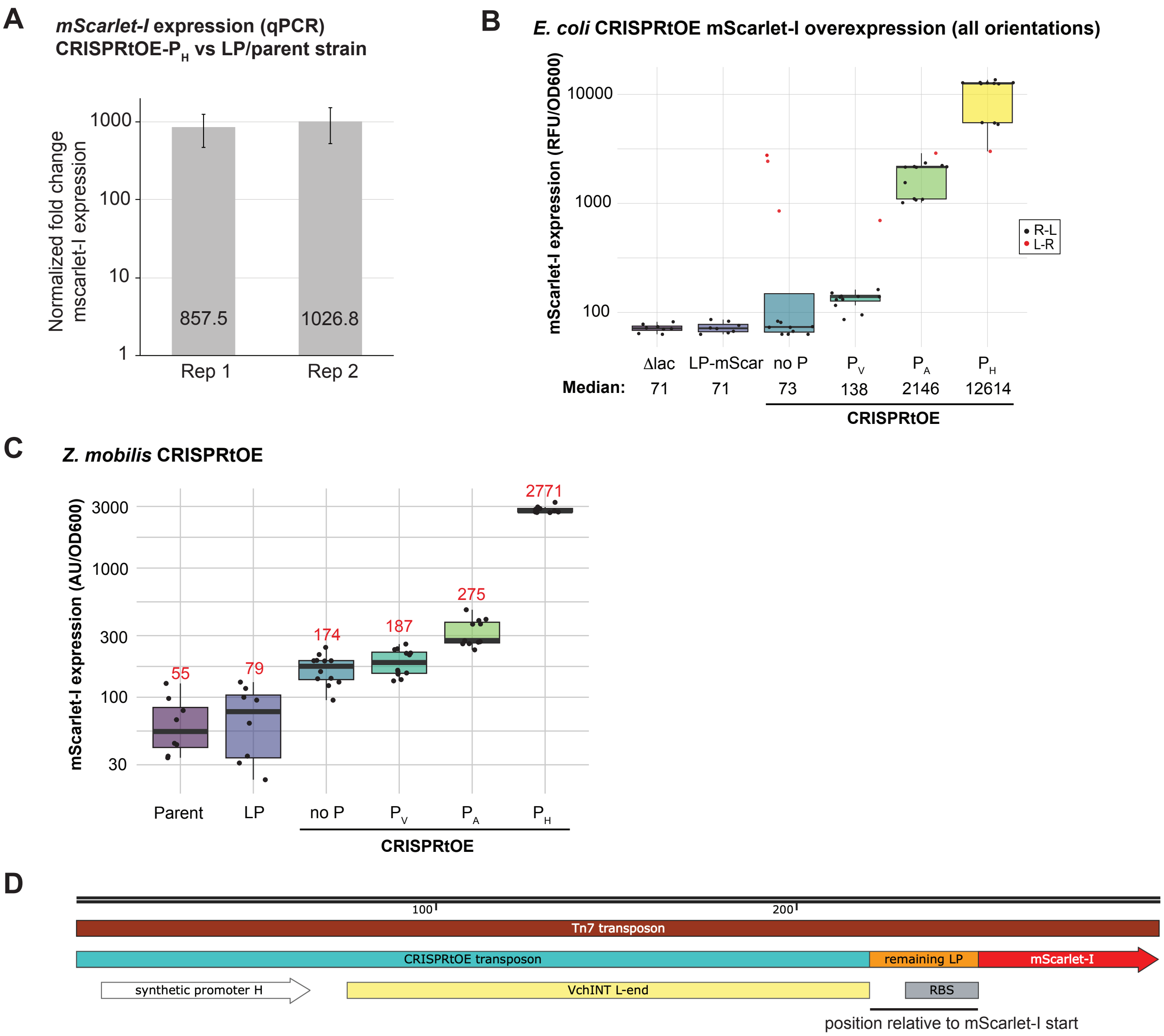

Position of CRISPRtOE transposon in Landing Pad relative to mScarlet-I start (LZ1 guide)

	# insertions analyzed	RL vs LR orientation		Position relative to ATG start (% of total RL insertions)									
	total RL and LR	%RL	%LR	-35	-34	-33	-32	-31	-30	-13	-2	+19	+108
Eco	48	87.5	12.5	21.4	2.4	71.4	0.0	0.0	4.8	0.0	0.0	0.0	0.0
Aba	12	100.0	0.0	0.0	0.0	66.7	0.0	0.0	0.0	8.3	8.3	8.3	8.3
Ecl	8	87.5	12.5	28.6	0.0	71.4	0.0	0.0	0.0	0.0	0.0	0.0	0.0
Kpn	10	90.0	10.0	22.2	0.0	77.8	0.0	0.0	0.0	0.0	0.0	0.0	0.0
Pae	12	91.7	8.3	36.4	0.0	63.6	0.0	0.0	0.0	0.0	0.0	0.0	0.0
Ppu	12	83.3	16.7	10.0	10.0	80.0	0.0	0.0	0.0	0.0	0.0	0.0	0.0
Son	11	100.0	0.0	36.4	9.1	45.5	0.0	0.0	9.1	0.0	0.0	0.0	0.0
Zmo	48	91.7	8.3	0.0	2.3	95.5	2.3	0.0	0.0	0.0	0.0	0.0	0.0

Figure S8

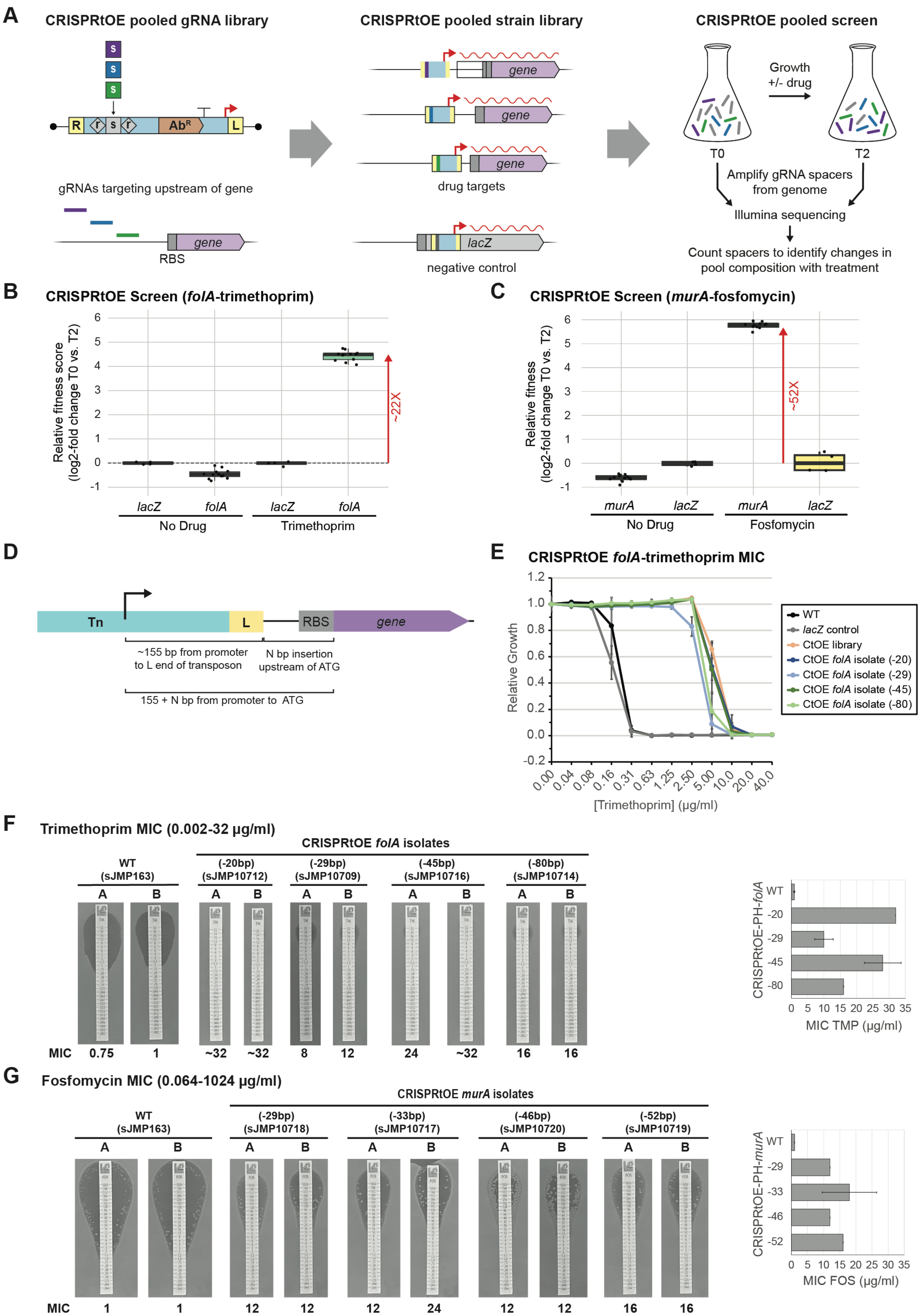
A
CRISPRtOE pooled gRNA library
S
S
S
R
r
s
r
Ab^R
L
gRNAs targeting upstream of gene
gene
RBS
CRISPRtOE pooled strain library
gene
gene
gene
drug targets
lacZ
negative control
CRISPRtOE pooled screen
Growth +/- drug
T0
T2
Amplify gRNA spacers from genome
Illumina sequencing
Count spacers to identify changes in pool composition with treatment
B
CRISPRtOE Screen (folA-trimethoprim)
Relative fitness score (log2-fold change T0 vs. T2)
6
5
4
3
2
1
0
-1
~22X
lacZ
folA
lacZ
folA
No Drug
Trimethoprim
C
CRISPRtOE Screen (murA-fosfomycin)
Relative fitness (log2-fold change T0 vs. T2)
6
5
4
3
2
1
0
-1
~52X
murA
lacZ
murA
lacZ
No Drug
Fosfomycin
D
Tn
L
RBS
gene
~155 bp from promoter to L end of transposon
N bp insertion upstream of ATG
155 + N bp from promoter to ATG
E
CRISPRtOE folA-trimethoprim MIC
Relative Growth
1.2
1.0
0.8
0.6
0.4
0.2
0.0
-0.2
0.00
0.04
0.08
0.16
0.31
0.63
1.25
2.50
5.00
10.0
20.0
40.0
[Trimethoprim] (µg/ml)
WT
lacZ control
CtOE library
CtOE folA isolate (-20)
CtOE folA isolate (-29)
CtOE folA isolate (-45)
CtOE folA isolate (-80)
F
Trimethoprim MIC (0.002-32 µg/ml)
CRISPRtOE folA isolates
WT (sJMP163)
(-20bp) (sJMP10712)
(-29bp) (sJMP10709)
(-45bp) (sJMP10716)
(-80bp) (sJMP10714)
A B A B A B A B A B
MIC 0.75 1 ~32 ~32 8 12 24 ~32 16 16
CRISPRtOE-PH-folA
WT
-20
-29
-45
-80
0 5 10 15 20 25 30 35
MIC TMP (µg/ml)
G
Fosfomycin MIC (0.064-1024 µg/ml)
CRISPRtOE murA isolates
WT (sJMP163)
(-29bp) (sJMP10718)
(-33bp) (sJMP10717)
(-46bp) (sJMP10720)
(-52bp) (sJMP10719)
A B A B A B A B A B
MIC 1 1 12 12 12 24 12 12 16 16
CRISPRtOE-PH-murA
WT
-29
-33
-46
-52
0 5 10 15 20 25 30 35
MIC FOS (µg/ml)

Figure S9

A Multiplex strain construction

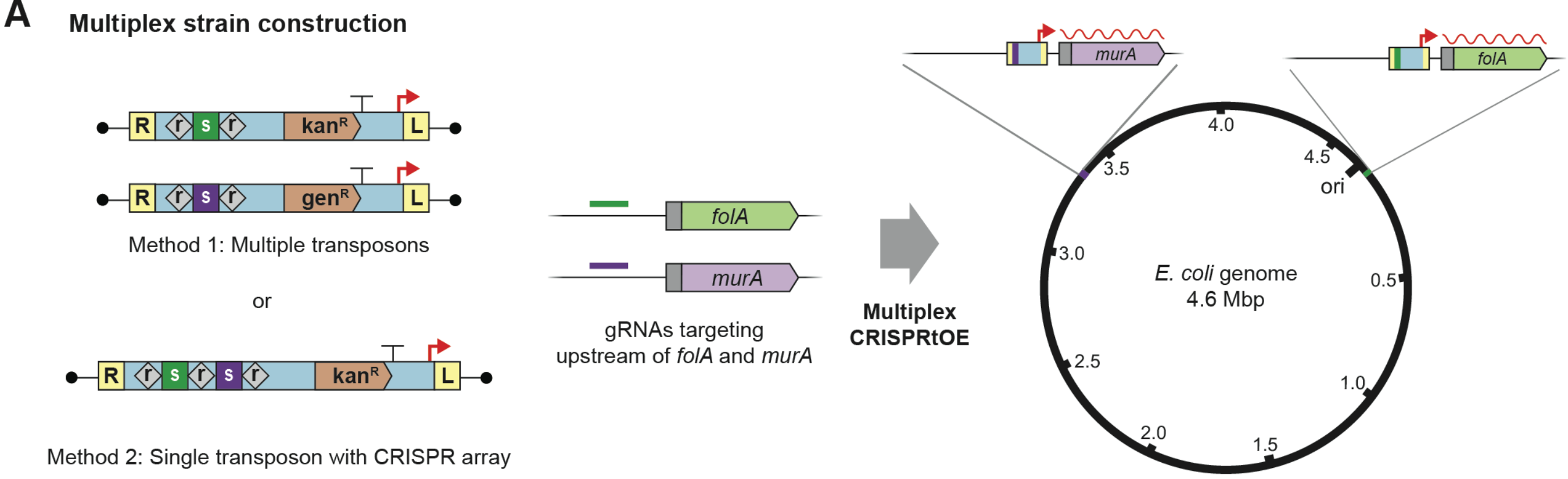

B Multiplex efficiency (*E. coli*)

Method	Transposon donor	crRNA	Conjugation	Selection	Insertion/genotype confirmed by PCR	Resistance phenotypes confirmed
1	Dual	1/donor	quad-parental	kan and gen	31/32 (97%)	4/4
2	Single	2/donor (array)	Tri-parental	kan	4/32 (12.5%)	4/4

C Trimethoprim MIC (0.002-32 µg/ml)

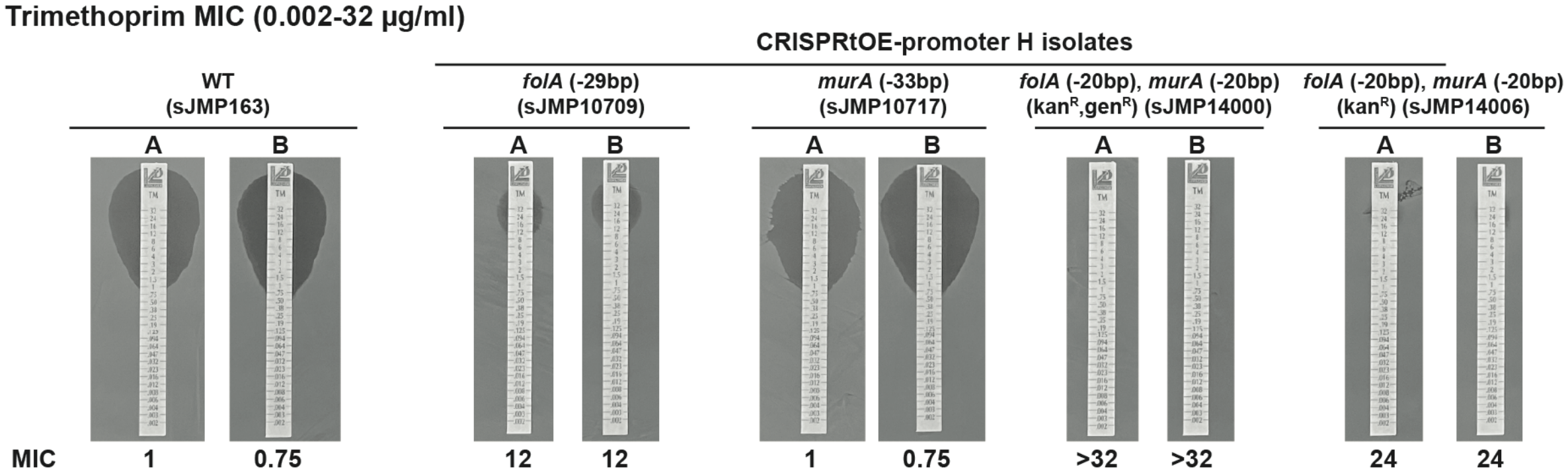

D Fosfomycin MIC (0.064-1024 µg/ml)

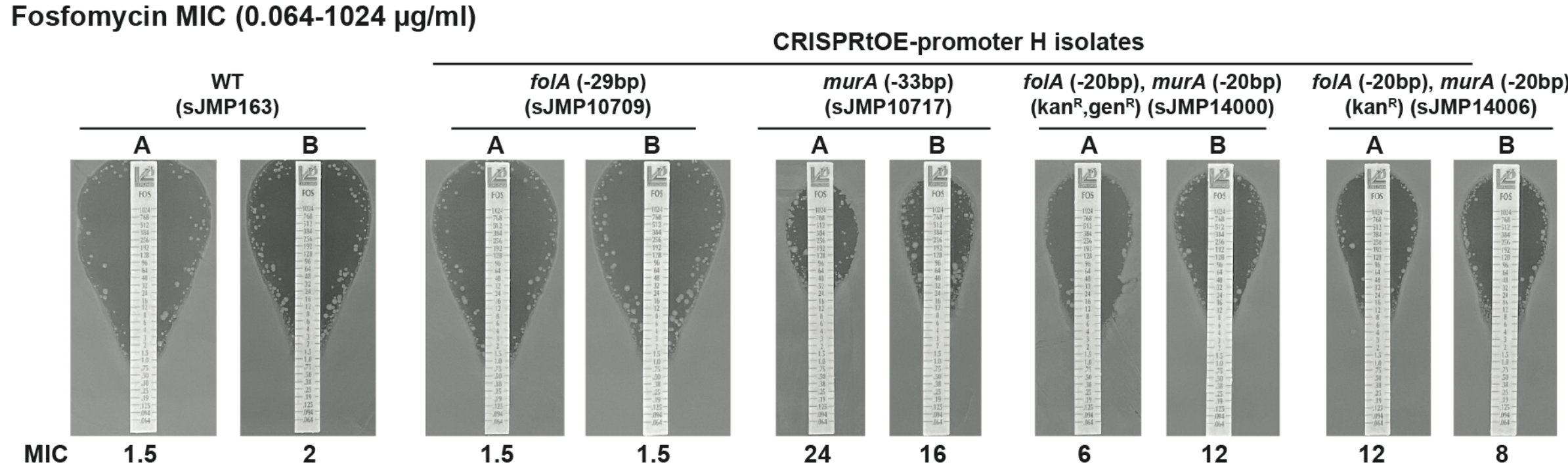

Supplement: Supplementary file 1 [file mbio.02340-25-s0001.pdf]
