# Supplementary material for: Targeted, genome-scale overexpression in Proteobacteria
Source: mBio. 2026 Sep 21;17(10):e02340-25. doi: 10.1128/mbio.02340-25 (PMC13712794; doi:10.1128/mbio.02340-25)
Supplement: Supplementary file 2 [file mbio.02340-25-s0002.pdf]

Figure S10

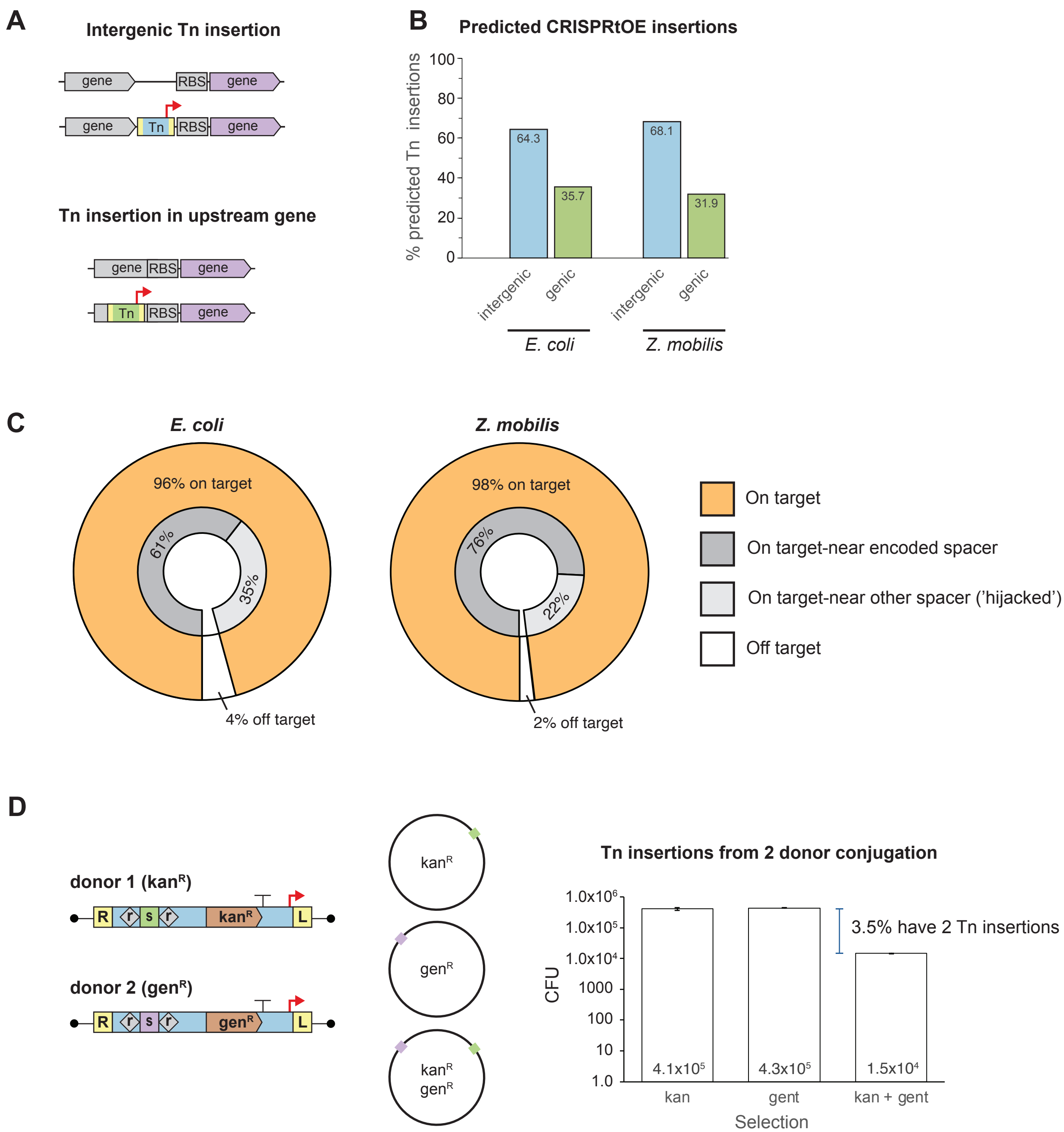
A
Intergenic Tn insertion
gene
RBS
gene
Tn
Tn insertion in upstream gene
B
Predicted CRISPRtOE insertions
% predicted Tn insertions
100
80
60
40
20
0
64.3
35.7
68.1
31.9
intergenic
genic
E. coli
Z. mobilis
C
E. coli
Z. mobilis
96% on target
61%
35%
4% off target
98% on target
76%
22%
2% off target
On target
On target-near encoded spacer
On target-near other spacer ('hijacked')
Off target
D
donor 1 (kanR)
donor 2 (genR)
R
r
s
kanR
genR
L
Tn insertions from 2 donor conjugation
1.0x10^6
1.0x10^5
1.0x10^4
1000
100
10
1.0
CFU
4.1x10^5
4.3x10^5
1.5x10^4
3.5% have 2 Tn insertions
kan
gent
kan + gent
Selection

Figure S11

A **Comparing Replicates With or Without Promoter H − Gene Medians**

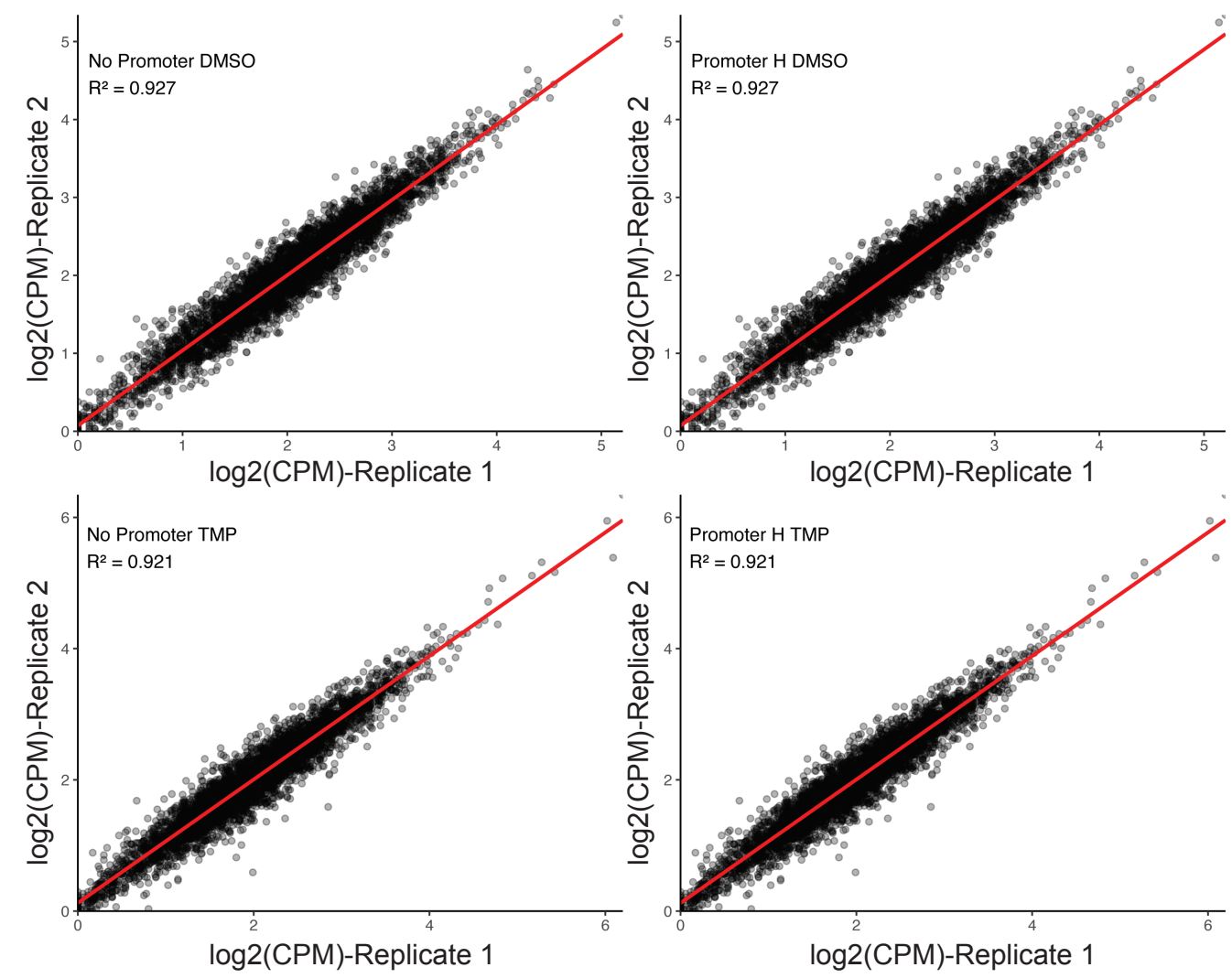

B **Comparing TMP vs DMSO With or Without Promoter H − Gene Medians**

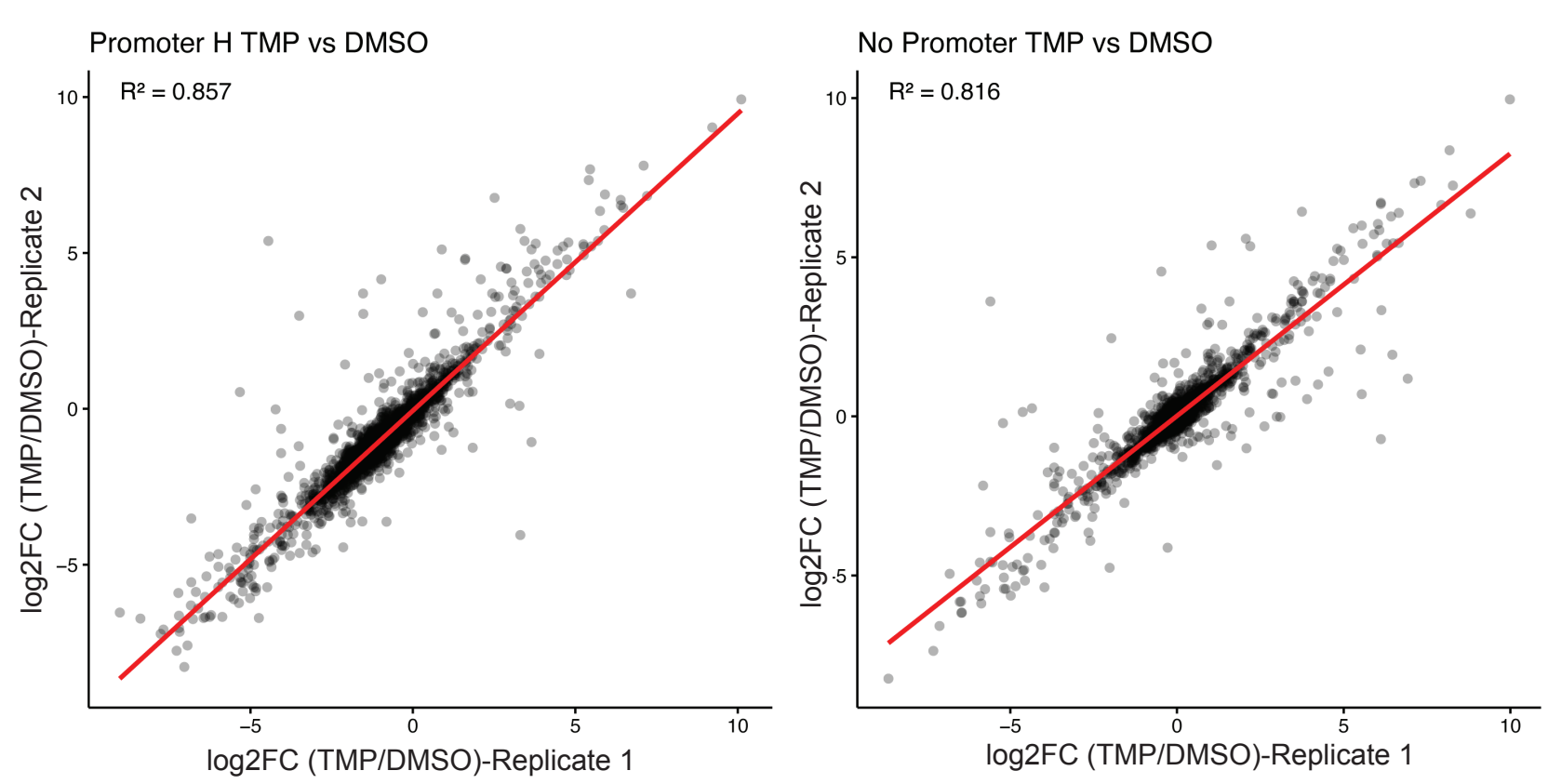

C **Comparing LFC TMP vs DMSO Gene Medians for CRISPRtOE With and Without Promoter**

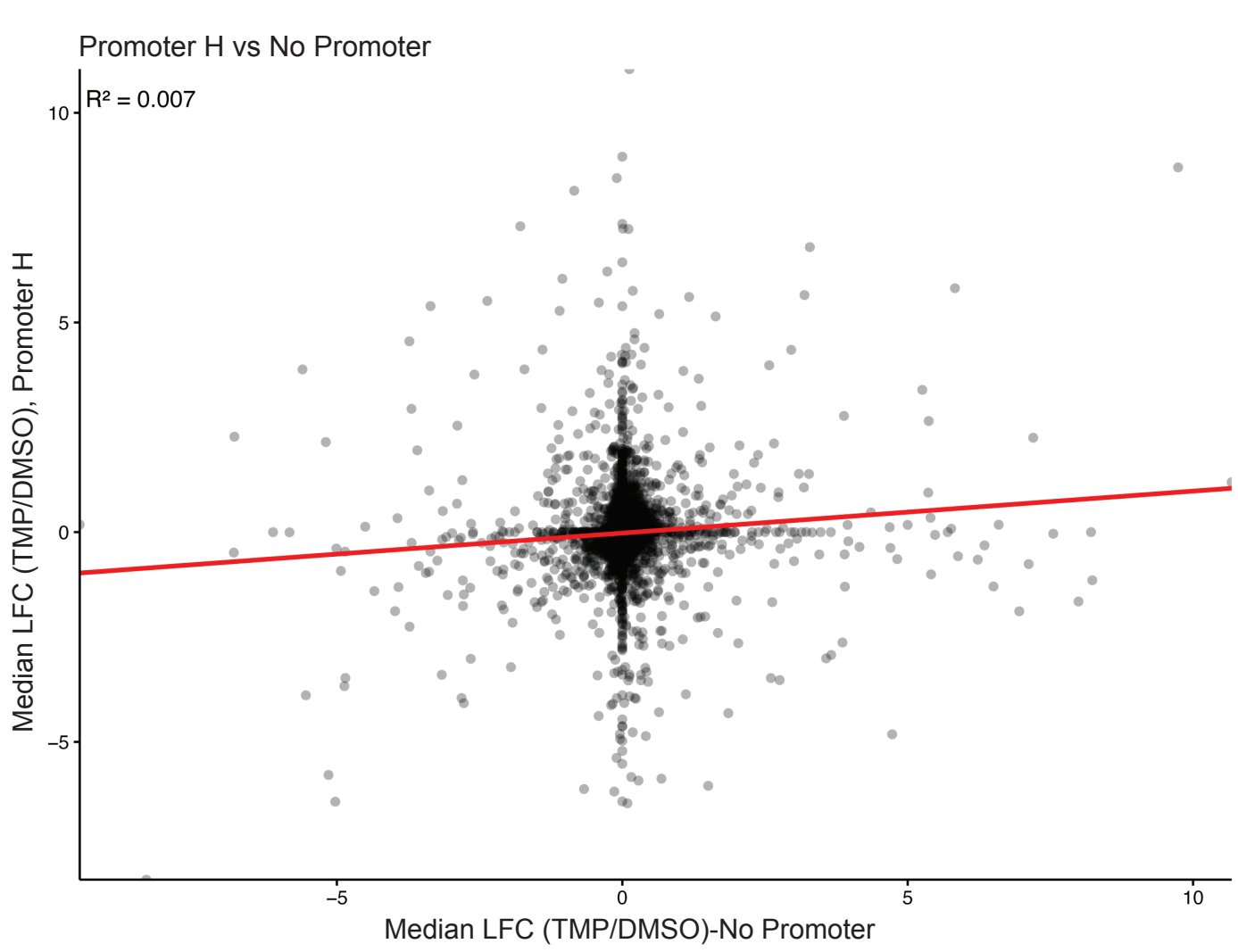

Figure S12

A

Log2 FC Gene Median TMP vs. DMSO treatment CRISPRtOE-Promoter H
Subset Number of Spacers vs All Spacers

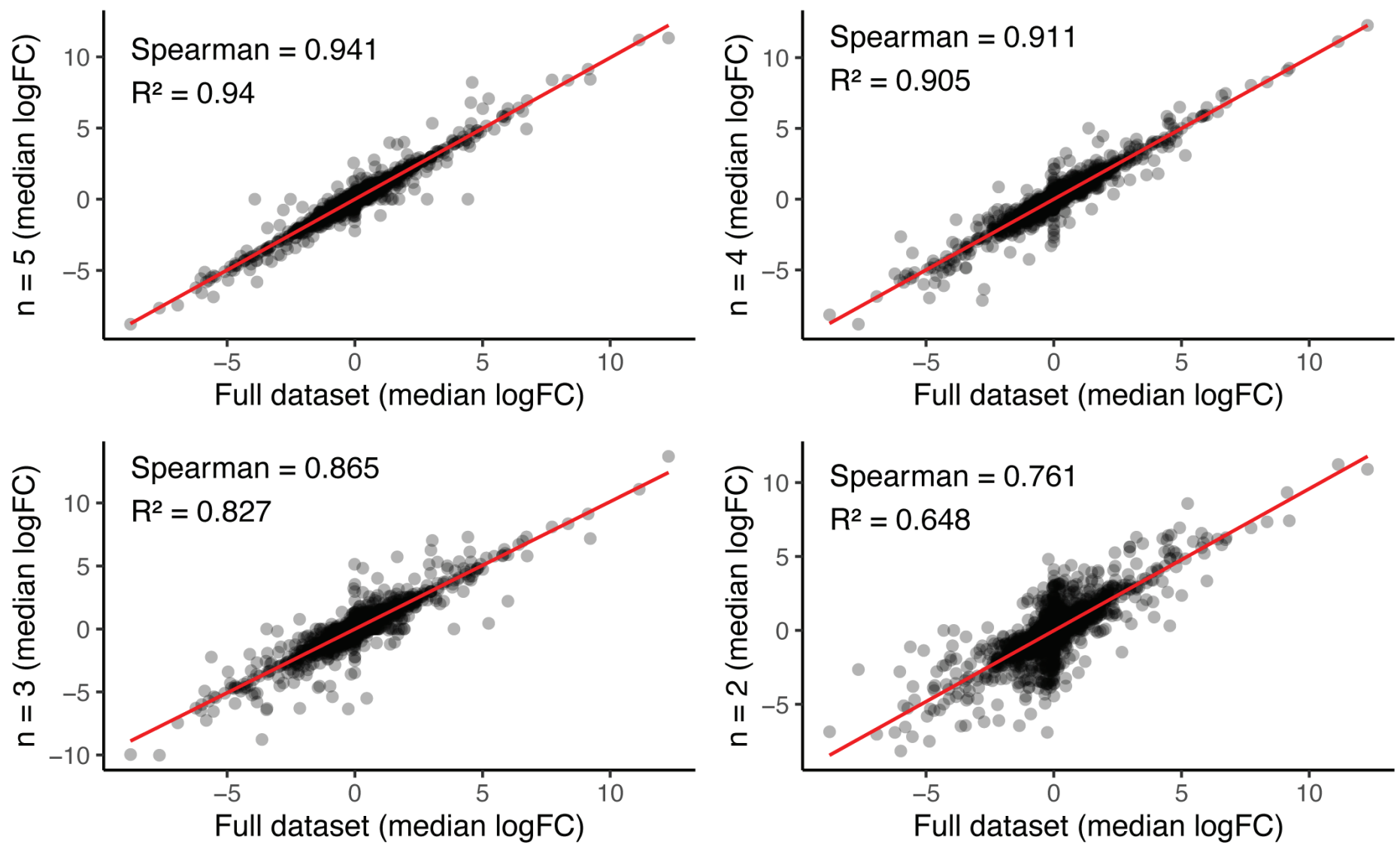

Figure S13

First Genes in Operon

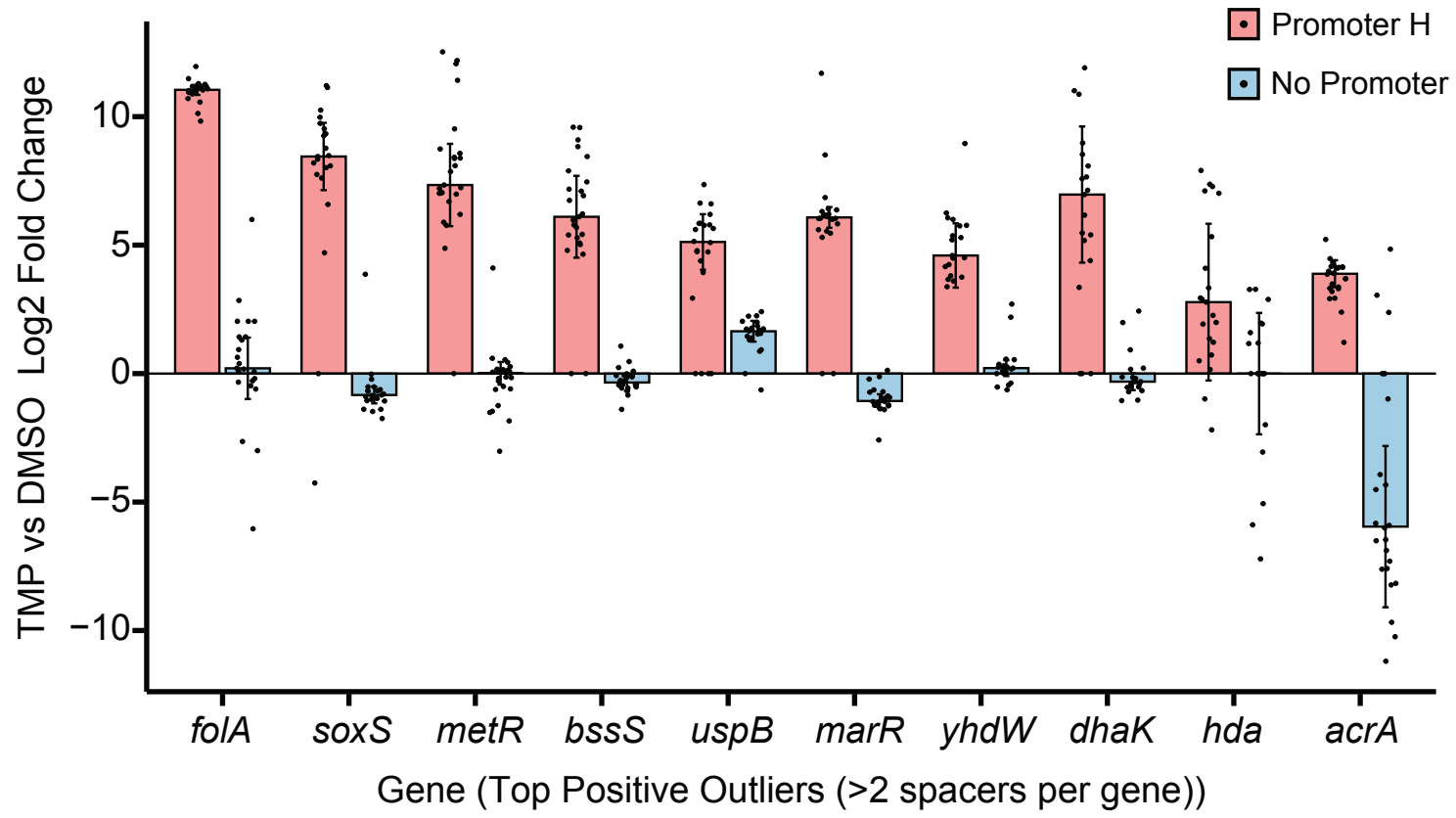

Figure S14

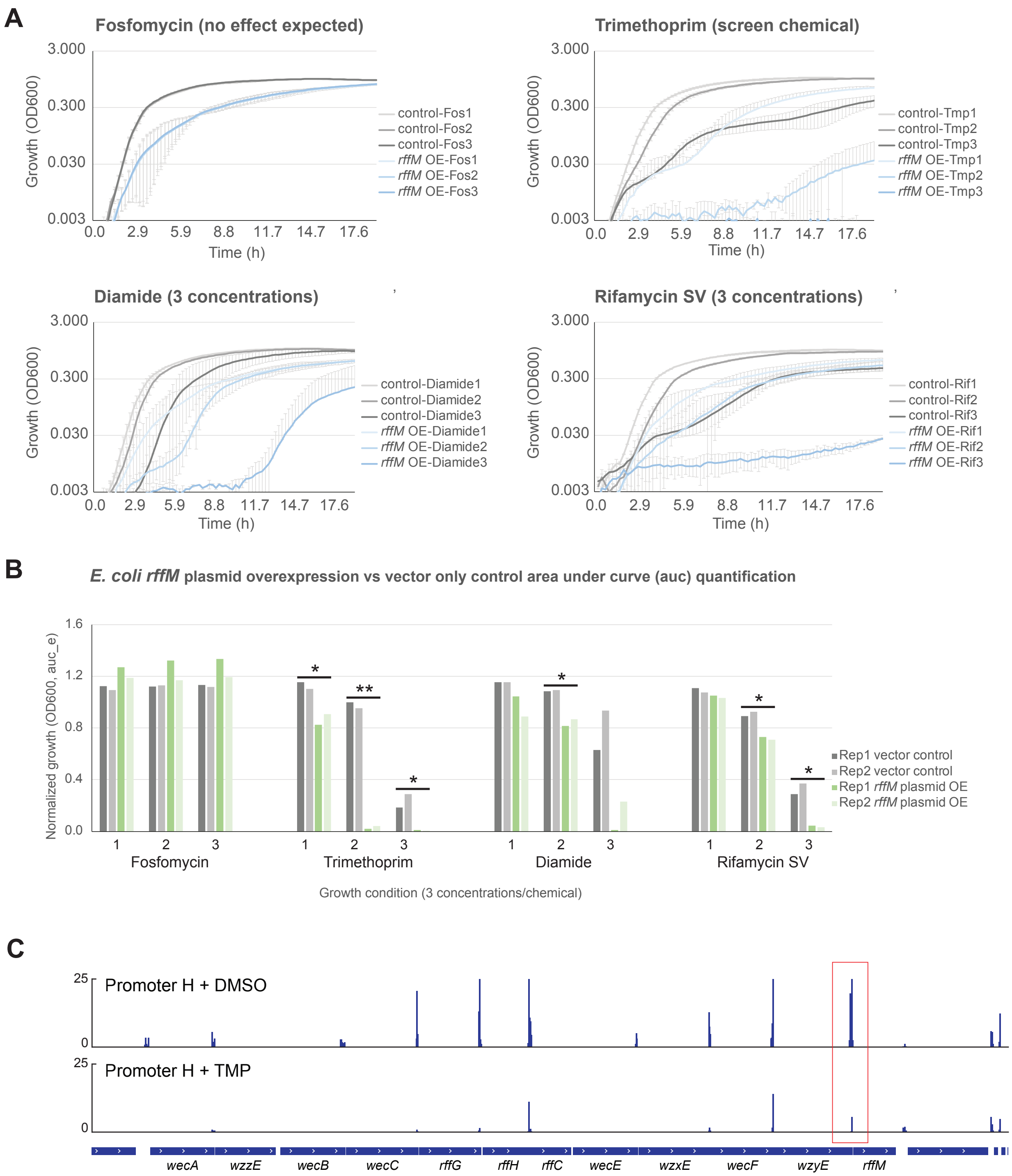
A
Fosfomycin (no effect expected)
Growth (OD600)
3.000
0.300
0.030
0.003
0.0
2.9
5.9
8.8
11.7
14.7
17.6
Time (h)
control-Fos1
control-Fos2
control-Fos3
rffM OE-Fos1
rffM OE-Fos2
rffM OE-Fos3
Trimethoprim (screen chemical)
control-Tmp1
control-Tmp2
control-Tmp3
rffM OE-Tmp1
rffM OE-Tmp2
rffM OE-Tmp3
Diamide (3 concentrations)
control-Diamide1
control-Diamide2
control-Diamide3
rffM OE-Diamide1
rffM OE-Diamide2
rffM OE-Diamide3
Rifamycin SV (3 concentrations)
control-Rif1
control-Rif2
control-Rif3
rffM OE-Rif1
rffM OE-Rif2
rffM OE-Rif3
B
E. coli rffM plasmid overexpression vs vector only control area under curve (auc) quantification
Normalized growth (OD600, auc_e)
1.6
1.2
0.8
0.4
0.0
Rep1 vector control
Rep2 vector control
Rep1 rffM plasmid OE
Rep2 rffM plasmid OE
1
2
3
Fosfomycin
Trimethoprim
Diamide
Rifamycin SV
Growth condition (3 concentrations/chemical)
C
25
0
Promoter H + DMSO
Promoter H + TMP
wecA
wzzE
wecB
wecC
rffG
rffH
rffC
wecE
wzxE
wecF
wzyE
rffM

Figure S15

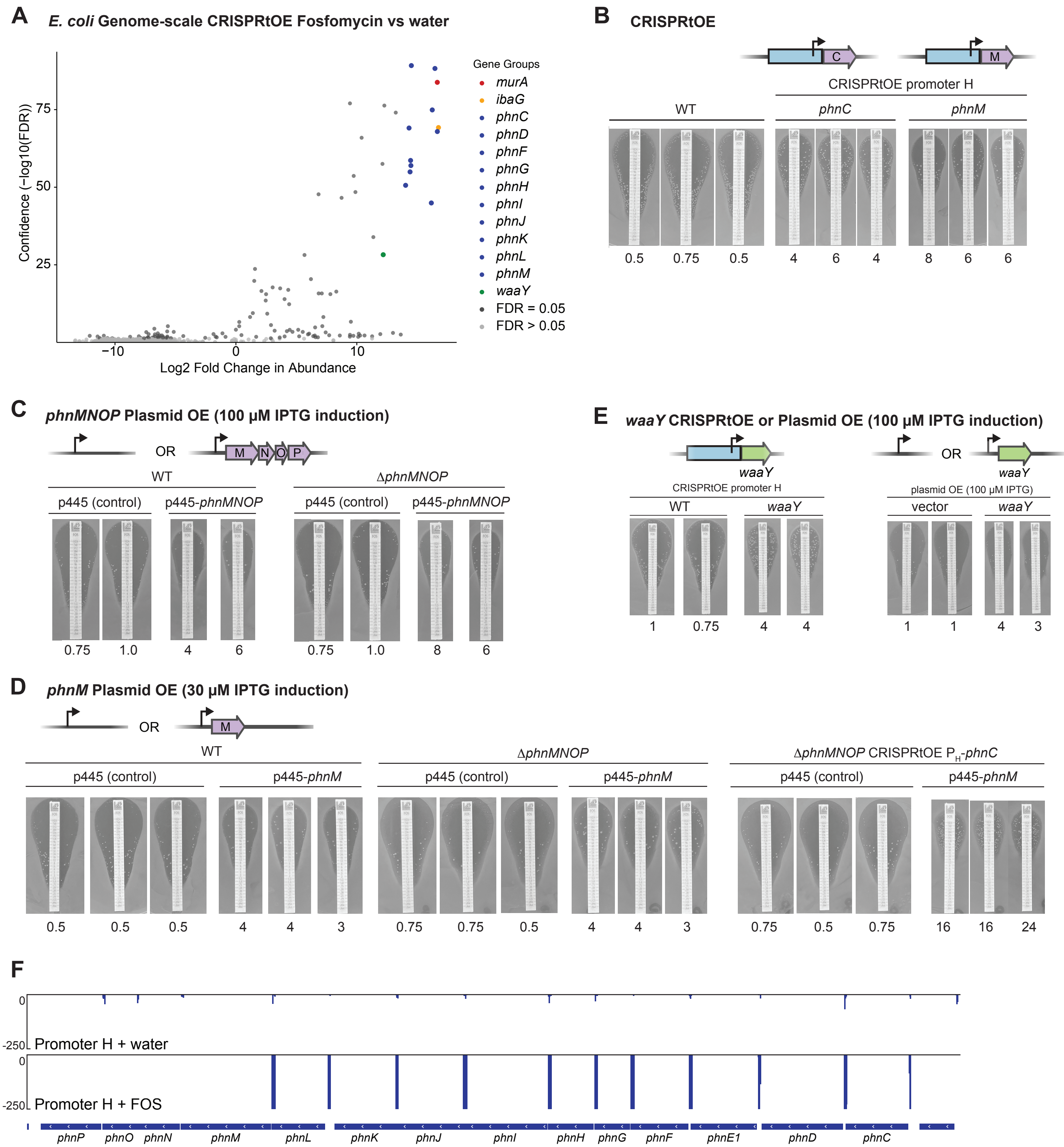
A
E. coli Genome-scale CRISPRtOE Fosfomycin vs water
Confidence (−log10(FDR))
Log2 Fold Change in Abundance
Gene Groups
murA
ibaG
phnC
phnD
phnF
phnG
phnH
phnI
phnJ
phnK
phnL
phnM
waaY
FDR = 0.05
FDR > 0.05
B
CRISPRtOE
CRISPRtOE promoter H
WT
phnC
phnM
0.5
0.75
0.5
4
6
4
8
6
6
C
phnMNOP Plasmid OE (100 µM IPTG induction)
OR
WT
ΔphnMNOP
p445 (control)
p445-phnMNOP
0.75
1.0
4
6
0.75
1.0
8
6
E
waaY CRISPRtOE or Plasmid OE (100 µM IPTG induction)
CRISPRtOE promoter H
plasmid OE (100 µM IPTG)
WT
waaY
vector
1
0.75
4
4
1
1
4
3
D
phnM Plasmid OE (30 µM IPTG induction)
ΔphnMNOP CRISPRtOE PH-phnC
p445-phnM
0.5
0.5
0.5
4
4
3
0.75
0.75
0.5
4
4
3
0.75
0.5
0.75
16
16
24
F
Promoter H + water
Promoter H + FOS
-250
phnP
phnO
phnN
phnM
phnL
phnK
phnJ
phnI
phnH
phnG
phnF
phnE1
phnD
phnC

Figure S16

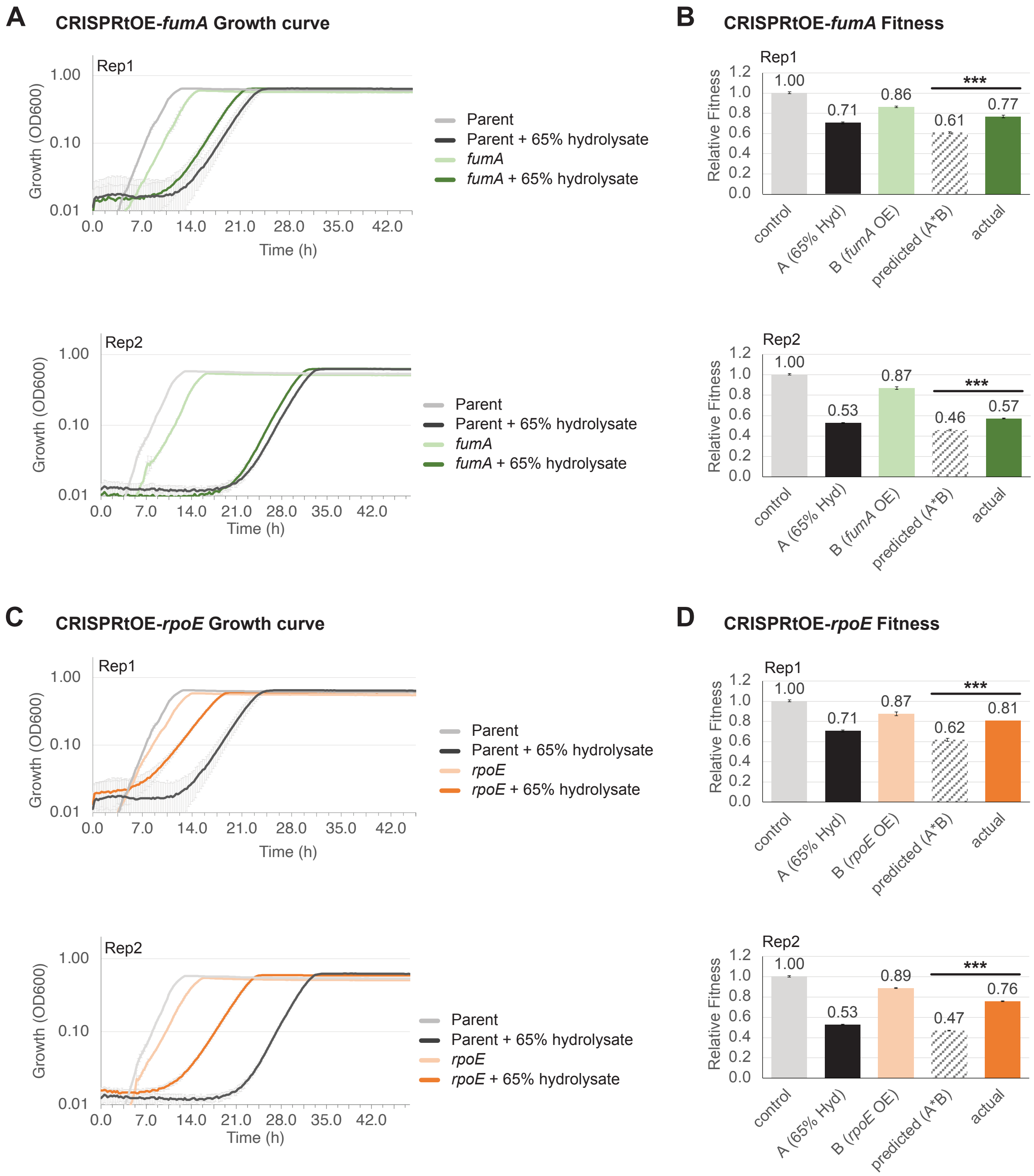
A
CRISPRtOE-*fumA* Growth curve
Rep1
Growth (OD600)
1.00
0.10
0.01
0.0
7.0
14.0
21.0
28.0
35.0
42.0
Time (h)
Parent
Parent + 65% hydrolysate
fumA
fumA + 65% hydrolysate
Rep2
Growth (OD600)
1.00
0.10
0.01
0.0
7.0
14.0
21.0
28.0
35.0
42.0
Time (h)
Parent
Parent + 65% hydrolysate
fumA
fumA + 65% hydrolysate
B
CRISPRtOE-*fumA* Fitness
Rep1
Relative Fitness
1.2
1.0
0.8
0.6
0.4
0.2
0.0
1.00
0.71
0.86
0.61
0.77
***
control
A (65% Hyd)
B (*fumA* OE)
predicted (A*B)
actual
Rep2
Relative Fitness
1.2
1.0
0.8
0.6
0.4
0.2
0.0
1.00
0.53
0.87
0.46
0.57
***
control
A (65% Hyd)
B (*fumA* OE)
predicted (A*B)
actual
C
CRISPRtOE-*rpoE* Growth curve
Rep1
Growth (OD600)
1.00
0.10
0.01
0.0
7.0
14.0
21.0
28.0
35.0
42.0
Time (h)
Parent
Parent + 65% hydrolysate
rpoE
rpoE + 65% hydrolysate
Rep2
Growth (OD600)
1.00
0.10
0.01
0.0
7.0
14.0
21.0
28.0
35.0
42.0
Time (h)
Parent
Parent + 65% hydrolysate
rpoE
rpoE + 65% hydrolysate
D
CRISPRtOE-*rpoE* Fitness
Rep1
Relative Fitness
1.2
1.0
0.8
0.6
0.4
0.2
0.0
1.00
0.71
0.87
0.62
0.81
***
control
A (65% Hyd)
B (*rpoE* OE)
predicted (A*B)
actual
Rep2
Relative Fitness
1.2
1.0
0.8
0.6
0.4
0.2
0.0
1.00
0.53
0.89
0.47
0.76
***
control
A (65% Hyd)
B (*rpoE* OE)
predicted (A*B)
actual

Figure S17

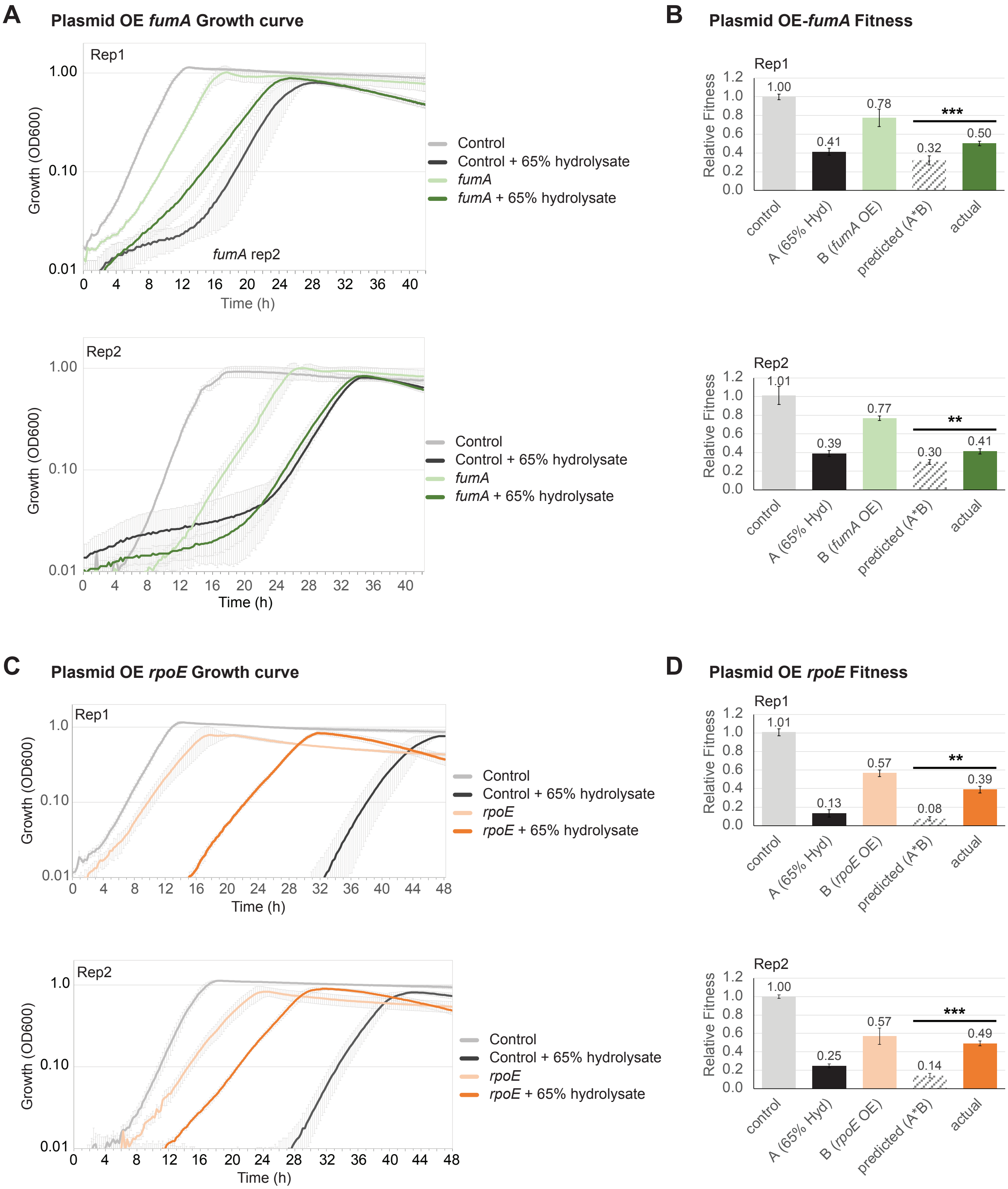
A
Plasmid OE fumA Growth curve
Rep1
Growth (OD600)
1.00
0.10
0.01
fumA rep2
Time (h)
Control
Control + 65% hydrolysate
fumA
fumA + 65% hydrolysate
Rep2
Growth (OD600)
Time (h)
Control
Control + 65% hydrolysate
fumA
fumA + 65% hydrolysate
B
Plasmid OE-fumA Fitness
Rep1
Relative Fitness
1.00
0.41
0.78
0.32
0.50
***
control
A (65% Hyd)
B (fumA OE)
predicted (A*B)
actual
Rep2
Relative Fitness
1.01
0.39
0.77
0.30
0.41
**
control
A (65% Hyd)
B (fumA OE)
predicted (A*B)
actual
C
Plasmid OE rpoE Growth curve
Rep1
Growth (OD600)
1.0
0.10
0.01
Time (h)
Control
Control + 65% hydrolysate
rpoE
rpoE + 65% hydrolysate
Rep2
Growth (OD600)
Time (h)
Control
Control + 65% hydrolysate
rpoE
rpoE + 65% hydrolysate
D
Plasmid OE rpoE Fitness
Rep1
Relative Fitness
1.01
0.13
0.57
0.08
0.39
**
control
A (65% Hyd)
B (rpoE OE)
predicted (A*B)
actual
Rep2
Relative Fitness
1.00
0.25
0.57
0.14
0.49
***
control
A (65% Hyd)
B (rpoE OE)
predicted (A*B)
actual
