# Supplementary material for: Targeted, genome-scale overexpression in Proteobacteria
Source: mBio. 2026 Sep 21;17(10):e02340-25. doi: 10.1128/mbio.02340-25 (PMC13712794; doi:10.1128/mbio.02340-25)
Supplement: Supplementary file 3 [file mbio.02340-25-s0003.docx]

**Banta et al. Supplemental Figure Legends**

**Figure S1.** Details of CRISPRt plasmids and *E. coli* GFP reporter strain. (A) Plasmid maps of CRISPRt-H helper and CRISPRt-T transposon plasmids. Both plasmids in the dual plasmid system have backbones with an R6K pir-dependent replication origin, an ampicillan resistance cassette, and conjugative transfer origin. The CRISPRt-H helper plasmid has expression cassettes for the minimal Vch CRISPR (Cas678) and Transposase (TnsABC, TniQ) machinery derived from pSpin-R(23). The CRISPRt-T transposon plasmid encodes a modified VchINT transposon derived from pSpin-R(23) which includes a guide RNA expression cassette with a modified BsaI cloning site and an antibiotic expression cassette and transcription terminators derived from CRISPRi plasmids(78). The antibiotic resistance cassette is flanked by XhoI sites to facilitate modification, six variants are shown. (B) Sequence of CRISPRt targeted transposition system guide RNA expression cassette including the BsaI cloning site for new guide RNAs. (C) Efficiency of obtaining CRISPRt transconjugants in *E. coli* using the *LZ1* and *gfp1* guides. Serial dilutions of three technical replicates of *LZ1* and *gfp1* CRISPRt assays were plated with and without selection (LB-kan or LB, respectively) and CFUs were compared; Efficiency of CRISPRt targeted transposition in *E. coli* with the *LZ1* guide was ~1 in 100 and with the *gfp1* guide was ~1 in 1000. **(**D) Position of the P_veg_-*sfgfp* expression cassette inserted between *yjaA* and *yjaB* on the *E. coli* chromosome.

**Figure S2.** CRISPRt disruption efficiency in *E. coli*. (A) Specificity of CRISPRt disruption of *lacZ* in *E. coli* measured by Tn-seq. Mapping of location of transposon insertion sites to the *E. coli* genome after CRISPRt targeted transposition the *LZ1* guide (216,801 reads). (B) Insertion position (bp downstream of protospacer) frequency of CRISPRt disruption of *gfp* and (C) *lacZ* in *E. coli* (R-L orientation). Percentage of on-target, R-L orientation insertions for all positions with > 0.01%. (D) Efficiency of CRISPRt disruption of *sfgfp* in *E. coli* using a fluorescence phenotypic assay. The *E. coli* *yjaA*-*sfgfp*-*yjaB* strain (sJMP2688) is green fluorescent whereas the *E. coli* WT strain (sJMP163) is not. Isolates from CRISPRt targeted transposition of *E. coli* *yjaA*-*sfgfp*-*yjaB* with the *gfp1* guide (selection for transconjugants on LB-kan plates) were assayed for fluorescence; 82/84 (97.6%) were white indicating disruption of *sfgfp*. (E) Efficiency of CRISPRt disruption of *lacZ* in *E. coli* using an X-gal phenotypic assay. The *E. coli* WT strain (sJMP163) can metabolize X-gal resulting in a blue colony color whereas the *E. coli* *lacZYA* deletion strain (sJMP3272) cannot metabolize X-gal resulting in a white colony color. Isolates from CRISPRt targeted transposition of *E. coli* WT with the *LZ1* guide (selection for transconjugants on LB-kan plates) were assayed for colony color; 90/90 (100%) were white indicating disruption of *lacZ*. (F) Frequency of co-integration of transposon-containing plasmid versus transposition of transposon only. *E. coli* CRISPRtOE libraries (sJMP10705 and sJMP10706) were serially diluted and plated on LB, LB-kan, and LB-carb in triplicate to determine total CFU, CFU with selection for transposon, CFU with selection for insertion of entire transposon-containing plasmid.

**Figure S3.** Transcription impacts efficacy of CRISPRt guides**.** (A) Schematic of guides in pooled *mScarlet-I* disruption experiment. Twenty-three guides matching protospacers located on top (T1-24) or bottom (B1-24) strand of the mScarlet-I encoding gene with a range of CN guides (see Table S5 for details). (B) Relative efficacy of CRISPRt *mScarlet-I* guides in a pooled screen targeting *mScarlet-I* expressed from a transposon in the *att*_Tn_*_7_* site on the *E. coli* chromosome. Relative efficacy is expressed as the log_2_-fold change of the frequency in the CRISPRt libraries (sJMP10519 and sJMP10520) vs. the frequency in the original plasmid construct libraries (sJMP10505 and sJMP10506). Average and standard deviation of two separate CRISPRt libraries (gentamicin^R^ and kanamycin^R^) containing all 46 guides. (C) Relative efficacy data ordered by guide position rather than efficacy shows no correlation between position and efficacy. (D) No correlation between PAM sequence (CA, CC, CG, CT) and efficacy (frequency of insertion) for 46 *mScarlet-I* targeting guides.

**Figure S4**. Transcription impacts efficacy of CRISPRt guides. (A) Schematic of *mScarlet-I* cassettes +/- promoter on the *E. coli* chromosome. The *mScarlet-I* expression cassette (red) with and without a promoter (orange) along with a spectinomycin resistance cassette (spt^R^, brown) are located on the *E. coli* chromosome just downstream of *glmS* and upstream of *pstS*. The *mScarlet-I* expression cassette is inserted in one of two orientations so that the direction of transcription is either in the same or the opposite direction of the leading strand of chromosomal DNA replication. (B) Relative efficacy of CRISPRt *mScarlet-I* guides in a pooled screen targeting *mScarlet-I* expressed on the *E. coli* chromosome in different orientations with and without transcription. Relative efficacy is expressed as the log2-fold change of the frequency in the CRISPRt libraries (sJMP10519-10520, sJMP10594-10595, sJMP10637-10640) vs. the frequency in the original plasmid construct libraries (sJMP10505 and sJMP10506) with average and standard deviation of two separate CRISPRt libraries (gentamicin^R^ and kanamycin^R^) containing all 46 guides shown.

**Figure S5.** Transcription impacts efficacy of CRISPRt guides, continued. (A) Relative efficacy of CRISPRt gRNAs in a pooled screen targeting *mScarlet-I* as in Figure S4 with average and standard deviation of two separate CRISPRt libraries (gentamicin^R^ and kanamycin^R^) containing all 46 guides shown on scatter plots. (B) Fluorescent phenotypes of 80 isolates from each of the *E. coli* CRISPRt *mScarlet-I* individual guide libraries (sJMP10641-10646) compared to *E. coli* strains with no *mScarlet-I* gene ((-) mScar, sJMP6999) or expressing *mScarlet-I* ((+) mScar, sJMP10205). Only 4/480 (0.83%) retained *mScarlet-I* levels equal to the parent strain indicating an off-target rate of <1% overall.

**Figure S6.** CRISPRtOE construct details. (A) Sequence alignment of CRISPRtOE promoters A, H, and V compared to a consensus *E. coli* σ^70^ promoter. Promoter A is based on the *lacUV5* promoter. Promoter H and V are synthetic promoters. Promoter H has an UP element highlighted in yellow. (B) Schematics of CRISPRtOE Tn*7*-like transposon constructs with four different outward facing promoter sequences (no promoter, and constitutive promoters A, H, and V; see Table S3 for plasmid details). Plasmids have been submitted to Addgene (ID 225832-225871). (C) Schematic of the chromosomally-encoded promoterless *mScarlet-I* reporter contained on a Tn*7* transposon inserted into the *att*_Tn_*_7_* site of *E. coli*.

**Figure S7.** Tunable overexpression of chromosomally located genes using CRISPRtOE.

(A) Quantification by qPCR of the *E. coli* CRISPRtOE-P_H_-*mScarlet-I* strain versus the promoterless 'Landing Pad' parent strain. Assay was repeated twice each with three biological replicates. *mScarlet-I* expression was normalized to expression of *rho*. (B) mScarlet-I fluorescence analysis of *E. coli* CRISPRtOE isolates (no promoter or synthetic promoters A, V, and H) compared to the parent (promoterless *mScarlet-I*) strain. As in Figure 2B except values are shown for all CRISPRtOE isolates (both R-L and L-R insertion orientations with R-L shown in black and L-R (flipped) shown in red). Error is expressed for the median value of 12 isolates in three replicate assays. (C) mScarlet-I fluorescence analysis of CRISPRtOE isolates of the Alphaproteobacterium *Zymomonas mobilis* (no promoter or synthetic promoters A, V, and H) compared to the parent (promoterless *mScarlet-I*) strain. Values are shown for isolates with on-target, R-L orientation CRISPRtOE insertions. Error is expressed for the median value of 8-12 isolates in three replicate assays. (D) Schematic showing insertion of CRISPRtOE transposon into the ‘Landing Pad’ upstream of *mScarlet-I*. Insertion position is variable (~45-55bp downstream of protospacer) resulting in a variable amount of the Landing Pad (containing the RBS) remaining upstream of the *mScarlet-I* coding sequence. Insertion positions and orientation of CRISPRtOE isolates in the eight species shown in Figure 2C. Insertion position is expressed relative to start of *mScarlet-I* coding sequence (equal to the remaining LP sequence). Table shading is proportional to the fraction of transposons existing in a specific orientation (green, ranging from 0-100%) or position from the *mScarlet-I* translation start site (blue, ranging from 0-95%).

**Figure S8.** CRISPRtOE *folA* and *murA* pooled library screen and MIC assays inform antibiotic mode of action. (A) Schematic of CRISPRtOE competitive growth assay. A pooled CRISPRtOE strain library was constructed by amplification of spacer sequences from a pooled oligo library and transfer onto the chromosome of a recipient strain. Insertion of the CRISPRtOE transposon is either upstream of genes of interest (12 guides/gene) or within the *lacZ* gene as a control gene (4 guides/gene) in *E. coli*. A CRISPRtOE pooled library was screened by culturing a CRISPRtOE pooled library and control library together for multiple generations in the presence or absence of a chemical of interest (trimethoprim (TMP) for the *folA* library and fosfomycin (FOS) for the *murA* library). The change in the composition of the pooled library before and after treatment was measured by amplification and NGS sequencing of the spacer sequences. (B) CRISPRtOE *folA* pooled library screen in TMP. Fitness of pooled strains overexpressing *folA* compared to the *lacZ* control strains in the absence or presence of TMP. (C) CRISPRtOE *murA* pooled library screen in FOS. (D) Schematic of insertion position for CRISPRtOE isolates. (E) MIC broth microdilution assay of four individual CRISPRtOE *folA* strains compared to WT, a CRISPRtOE *lacZ* isolate, and the CRISPRtOE *folA* pooled library in the presence of TMP (0.04-20 µg/ml). Error is expressed for 3 separate assays. (F) MIC test strip assays (trimethoprim, 0.002-32 µg/ml) comparing WT and four distinct CRISPRtOE *folA* isolates quantified as average and standard deviation of two assays. (G) MIC test strip assays (fosfomycin, 0.064-1024 µg/ml) comparing WT and four distinct CRISPRtOE *murA* isolates quantified as average and standard deviation of two assays.

**Figure S9.** Multiplexed CRISPRtOE strain construction. (A) Schematic of two methods of multiplexed CRISPRtOE strain construction in *E. coli*. Method 1 utilizes quad-parental mating with two CRISPRtOE transposon donors with different antibiotic resistance genes while Method 2 utilizes tri-parental mating with a single transposon donor with a single antibiotic resistance gene and a CRISPR array encoding multiple guides. Guides upstream of *folA* or *murA* that were previously shown to be effective at conferring TMP or FOS resistance (see Figure S8) were used to simultaneously guide the CRISPRtOE-promoter H Tn insertion into the genome of *E. coli*. (B) Efficiency of multiplexed insertion of CRISPRtOE-promoter H-*folA* and CRISPRtOE-promoter H-*murA* as determined by PCR of 32 isolates. Increased TMP and FOS resistance of 4 PCR-confirmed isolates was confirmed by disc diffusion assays. (C) TMP resistance of 1 sequence-confirmed isolate from each of the two methods was compared to WT and previously isolated single isolates (either CRISPRtOE-promoter H-*folA* or CRISPRtOE-promoter H-*murA*. (D) FOS resistance of 1 sequence-confirmed isolate from each of the two methods was compared to WT and previously obtained single isolates (either CRISPRtOE-promoter H-*folA* or CRISPRtOE-promoter H-*murA*.

**Figure S10.** Tn insertion in *E.coli* and *Z. mobilis* CRISPRtOE genome-scale libraries. (A) Schematic of two possible transposon insertion positions: intergenic/entirely between genes or Tn inserted in front of a gene disrupts the end of the gene upstream of it. (B) Percentage of insertions in the *E. coli* and *Z. mobilis* genome-scale CRISPRtOE libraries that are intergenic versus in an upstream gene. (C) Percentage of Tn insertions that are on- (orange) or off- (white) target and percentage on on-target Tn insertions whose position corresponds to the encoded spacer (medium gray) or is instead near the predicted insertion position based on another 'hijacked' spacer (light gray). (D) Multiplex CRISPRtOE experiment to estimate number of strains that may have multiple Tn insertions. Quad-parental mating was performed with two CRISPRtOE transposon donors with different antibiotic resistance genes (kan^R^ or gen^R^) that were previously shown to effectively target insertion upstream of *folA* or *murA* in *E. coli.* Transconjugants selected on kan, gen, or kan and gen were quantified in three technical replicates and the difference between the number of transconjugants with a single antibiotic resistance phenotype vs. a dual antibiotic resistance phenotype was calculated.

**Figure S11.** Genome-scale CRISPRtOE reproducibility. (A) Comparison of two biological replicates of CRISPRtOE screen data at the gene level (CPM = counts per million). Either CRISPRtOE library with Promoter H or no promoter with either TMP or DMSO control treatment showing correlation (R^2^ = ~0.9).(B) Comparison of two biological replicates of the log2FC (FC = fold change) of TMP versus DMSO control treatment for either the CRISPRtOE library with Promoter H or no promoter showing correlation (R^2^ = ~0.8). (C) Comparison of the median LFC (Log Fold Change) of TMP versus DMSO control treatment for the CRISPRtOE Promoter H versus no promoter libraries showing no correlation (R^2^ = ~0.007).

**Figure S12.** Robustness of CRISPRtOE with fewer analyzed guides. Scatterplots comparing all spacers measured in our study to subsets of 5, 4, 3, or 2 randomly selected spacers per gene.

**Figure S13.** *E. coli* CRISPRtOE TMP screen top positive outlier genes considering only the first gene in the operon. Genes must have at least a 4-fold change in the CRISPRtOE Promoter H data and show a 4-fold difference in a comparison between Promoter H (red) and no promoter (blue) to be considered. Dots represent unique insertions. Error bars represent standard deviation (SD).

**Figure S14.** Growth assay of strains overexpressing *rffM* from a plasmid in additional chemical conditions using a Biolog phenotypic array plate PM16 comprised of 24 chemicals with 4 concentrations each. (A) Growth curves of are shown for CRISPRtOE screen chemicals FOS (no response expected) and TMP (response expected), and of two additional chemicals that had a significant response (diamide and rifamycin) for 3 concentrations. Error bars represent SD of duplicate assays. (B) Quantification of area under the curve (auc_e) of growth assays shown in panel A using GrowthCurver. Duplicate assays are shown (Rep1 and Rep2). Three concentrations are shown. Significance was determined by a t test (≤0.05(*), ≤0.01(**). Biolog PM16 compounds tested: cefotaxime, phosphomycin, 5-chloro-7-iodo-8-hydroxyquinoline, norfloxacin, sulfanilamide, trimethoprim, dichlofluanid, protamine sulphate, cetylpyridinium chloride, 1-chloro-2,4-dinitrobenzene, diamide, cinoxacin, streptomycin, 5-azacytidine, rifamycin SV, potassium tellurite, sodium selenite, aluminum sulphate, chromium chloride, ferric chloride, L-glutamic-g-hydroxamate, glycine hydroxamate, chloroxylenol, sorbic acid. (C) CRISPRtOE insertion frequency map at the *wec-rff* locus. The scale was limited to 25 counts to make the *rrfM* signal in TMP visually apparent.

**Figure S15.** *E. coli* genome scale CRISPRtOE screen with fosfomycin. (A) Volcano plot of gRNA spacer counts in fosfomycin (FOS) versus water control. Each dot represents a strain with a spacer targeting upstream of a gene. Those that are not statistically significant (false discovery rate (FDR) > 0.05) are shown in light grey and significant hits (FDR ≤ 0.05) are shown in dark gray. Several outliers are colored: *murA* (red), *ibaG* (yellow), *phnC-M* (blue), *waaY* (green). (B) MIC test strip (Liofilchem) assay (3 technical replicates) showing resistance to FOS of individual CRISPRtOE-Promoter H strains overexpressing *phnC* or *phnH* compared to WT. (C) MIC test strip (Liofilchem) assay (3 technical replicates) showing resistance to FOS of strains overexpressing *phnMNOP* from a plasmid with an IPTG-inducible promoter in WT or a Δ*phnMNOP* deletion strain. (D) MIC test strip (Liofilchem) assay (3 technical replicates) showing resistance to FOS of strains overexpressing *phnM* from a plasmid with an IPTG-inducible promoter in WT, Δ*phnMNOP* deletion, or Δ*phnMNOP* deletion plus CRISPRtOE-Promoter H-*phnC* overexpression strain. (E) MIC test strip (Liofilchem) assay (2 technical replicates) showing resistance to FOS of strains overexpressing *waaY* using CRISPRtOE-Promoter H (left) or from a plasmid with an IPTG-inducible promoter (right). (F) CRISPRtOE insertion frequency map at the *phn* locus. The scale was limited to 250 counts to make signals in the water only control visually apparent.

**Figure S16.** Fitness of *Z*. *mobilis* CRISPRtOE-Promoter H *fumA* and *rpoE* strains. (A) Growth assay of individually constructed CRISPRtOE-Promoter H *fumA* strains versus the parent strain in 65% hydrolysate or ZRDM control. Error bars represent standard deviation (SD) of three independant cultures. (B) Relative fitness of strains shown in panel (A). Growth was quantified by calculating the area under the curve (auc_e) using GrowthCurver. Fitness was normalized to control condition (parent strain in ZRDM). Significance was determined by a t test (≤0.005(***). (C) Growth assay of individually constructed CRISPRtOE-Promoter H *rpoE* strains versus the parent strain in 65% hydrolysate or ZRDM control. Error bars represent standard deviation (SD) of three independant cultures. (D) Relative fitness of strains shown in panel (C). Growth was quantified by calculating the area under the curve (auc_e) using GrowthCurver. Fitness was normalized to control condition (parent strain in ZRDM). Significance was determined by a t test (≤0.005(***).

**Figure S17.** Fitness of *Z*. *mobilis* strains overexpressing *fumA* and *rpoE* from a plasmid. (A) Growth assay of *fumA* plasmid overexpression strains versus a strain with an empty plasmid in 65% hydrolysate or ZRDM control. Error bars represent standard deviation (SD) of three independant cultures. (B) Relative fitness of strains shown in panel (A). Growth was quantified by calculating the area under the curve (auc_e) using GrowthCurver. Fitness was normalized to control condition (parent strain in ZRDM). Significance was determined by a t test (<0.01(**), ≤0.005(***). (C) Growth assay of *rpoE* plasmid overexpression strains versus a strain with an empty plasmid in 65% hydrolysate or ZRDM control. Error bars represent standard deviation (SD) of three independant cultures. (D) Relative fitness of strains shown in panel (C). Growth was quantified by calculating the area under the curve (auc_e) using GrowthCurver. Fitness was normalized to control condition (parent strain in ZRDM). Significance was determined by a t test (<0.01(**), ≤0.005(***).
